# Inference of alternative splicing from RNA-Seq data with probabilistic splice graphs

**DOI:** 10.1093/bioinformatics/btt396

**Published:** 2013-07-11

**Authors:** Laura H. LeGault, Colin N. Dewey

**Affiliations:** ^1^Department of Computer Sciences and ^2^Department of Biostatistics and Medical Informatics, University of Wisconsin, Madison, WI 53706, USA

## Abstract

**Motivation:** Alternative splicing and other processes that allow for different transcripts to be derived from the same gene are significant forces in the eukaryotic cell. RNA-Seq is a promising technology for analyzing alternative transcripts, as it does not require prior knowledge of transcript structures or genome sequences. However, analysis of RNA-Seq data in the presence of genes with large numbers of alternative transcripts is currently challenging due to efficiency, identifiability and representation issues.

**Results:** We present RNA-Seq models and associated inference algorithms based on the concept of probabilistic splice graphs, which alleviate these issues. We prove that our models are often identifiable and demonstrate that our inference methods for quantification and differential processing detection are efficient and accurate.

**Availability:** Software implementing our methods is available at http://deweylab.biostat.wisc.edu/psginfer.

**Contact:** cdewey@biostat.wisc.edu

**Supplementary information:** Supplementary data are available at *Bioinformatics* online.

## 1 INTRODUCTION

An important aspect of eukaryotic molecular biology is the fact that a single gene can give rise to a wide variety of transcripts as a result of pre-mRNA *alternative processing* (AP) events. AP events result from the phenomena of alternative splicing and alternative polyadenylation sites, which can give rise to different mRNAs from the same pre-mRNA, and alternative transcription start sites, which can lead to different pre-mRNAs produced from the same gene locus. The alternative transcripts resulting from these processes are important for development and have also been implicated in disease (Matlin *et al.*, 2005). Recent studies have shown that >90% of human genes (Wang *et al.*, 2008) and 60% of *Drosophila* genes (Graveley *et al.*, 2011) are alternatively spliced, indicating that this phenomenon is the rule rather than the exception.

The relatively recent technology of RNA-Seq is revolutionizing the way alternative transcripts are identified and quantified (Wang *et al.*, 2009). Unlike microarrays, which can only measure what their probes are designed to detect, RNA-Seq allows for the detection of novel splice junctions and exonic sequences [e.g. Trapnell *et al.* (2009)]. In fact, RNA-Seq is now commonly being used to perform *de novo* transcriptome assembly (Grabherr *et al.*, 2011; Robertson *et al.*, 2010), a powerful approach for studying gene expression in species without sequenced genomes.

In this article, we present a novel approach to the tasks of alternative transcript quantification and differential processing (DP) detection given RNA-Seq data and fixed gene structures, which may be provided by an existing genome annotation or predicted from the data via transcriptome assembly. Our approach is based on a class of models that were first independently described in the context of expressed sequence tag (EST) analysis (Chang *et al.*, 2005; Jenkins *et al.*, 2006). We refer to these models as *probabilistic splice graphs* (PSGs). PSGs build on the concept of *splice graphs* (Heber *et al.*, 2002), structures that compactly represent the possible isoforms of a gene, given a set of known exon boundaries and splice junctions. The primary contribution of this work is efficient methodology for applying PSGs to RNA-Seq data, which are more powerful than ESTs for quantifying alternative splicing owing to larger numbers of reads, but more challenging to analyze owing to shorter read lengths.

In general, PSGs provide a statistical framework for representing dependencies between AP events. The existence of dependencies, or lack thereof, between AP events is currently an active area of research. Several genes have been found to have independent splicing events (Emerick *et al.*, 2006; Neves *et al.*, 2004), whereas others suggest that dependencies exist (Emerick *et al.*, 2006; Fededa *et al.*, 2005). However, there is currently not enough full-length cDNA sequence data to determine if there are widespread dependencies between AP events. As we will discuss, our PSG-based RNA-Seq analysis methods can be configured to model and detect such dependencies, when they exist.

When processing events are largely independent of each other, a PSG allows for a compact statistical model of the frequencies of a gene’s isoforms. Our RNA-Seq analysis methods take advantage of this strength of PSGs to address a number of challenges posed by alternative splicing that are not thoroughly dealt with by previous methods. All of these challenges are rooted by the facts that (i) RNA-Seq reads identify only a small fraction of the transcript from which they are derived and (ii) alternative transcripts from the same gene typically share a large amount of sequence.

A first challenge is that a gene may have an exponential number of alternative splice forms, which makes quantification of individual isoforms extremely difficult. An exponential blowup in the number of possible isoforms occurs when multiple domains of a gene are subject to alternative splicing, each of which is spliced independently of the others. For example, the *Drosophila* gene *Dscam* is famous for having four alternatively spliced domains that result in over 38 000 possible isoforms (Schmucker *et al.*, 2000).

**Fig. 1.**
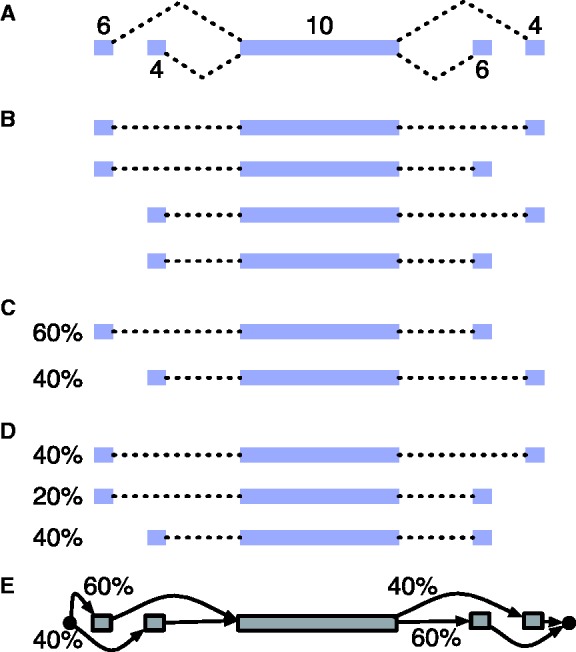
An example gene for which an explicit model of all possible isoform frequencies is not identifiable, whereas a PSG model for the gene is identifiable, given RNA-Seq reads. We assume that the RNA-Seq fragments are shorter than the middle exon and thus that reads from a fragment identify at most one splice junction. (**A**) The gene model with levels of coverage by RNA-Seq reads indicated above each exon. (**B**) The four possible isoforms of the gene. (**C**) and (**D**) give two (of infinitely many) possible isoform abundances that explain the observed RNA-Seq read coverages equally well. (**E**) The exon graph PSG for the gene, which is identifiable given this data (the unique ML parameters are above each edge), assuming the exon sequences are relatively unique

Second, models for quantifying full-length isoforms with single-end RNA-Seq data are often not identifiable for genes with many alternative splice forms (Hiller *et al.*, 2009; Lacroix *et al.*, 2008). For a model to be identifiable, different parameter values for the model must give rise to distinct probability distributions over possible datasets. The practical disadvantage of having a non-identifiable model is that for a given dataset (e.g. RNA-Seq reads), there may be multiple possible parameter settings (e.g. transcript abundances) that explain the data equally well. Figure 1 provides a simple example of a gene for which the frequencies of its four possible isoforms are not identifiable given typical RNA-Seq data. In theory, paired-end data can eliminate this issue (Lacroix *et al.*, 2008); however, in practice, paired-end data are derived from short size-selected fragments that provide local information similar to that of longer single-end data.

A third challenge for the analysis of RNA-Seq data in the presence of alternative splicing is that both *de novo* and genome-guided transcriptome assemblers often do not have enough information to output full-length transcripts for genes that have splice variants. For example, if a gene has two alternatively spliced domains spaced sufficiently far apart such that no reads (or paired-end reads) span both domains (as is the case in Fig. 1), then it may be impossible to determine which combinations of splice events are found in that gene’s isoforms. Faced with this challenge, current assemblers are either choosing a minimal set of isoforms (Li *et al.*, 2011a, b; Trapnell *et al.*, 2010; Xia *et al.*, 2011) or reporting all possible isoforms that are compatible with the data (Grabherr *et al.*, 2011; Guttman *et al.*, 2010). The former strategy risks underestimating the number of true isoforms, particularly when non-identifiability is an issue. The latter strategy is unlikely to omit true isoforms, but may result in the reporting of exponential numbers of isoforms, the abundances of which are likely not identifiable. Both strategies may result in the reporting of isoform structures that do not exist.

As we will show, our novel application of the PSG modeling framework to RNA-Seq analysis alleviates all three of these issues. First, we present an efficient Expectation-Maximization (EM)-based algorithm for estimating the maximum *a posteriori* (MAP) parameters of a PSG, given single or paired-end RNA-Seq data. The estimation of PSG parameters is equivalent to the alternative transcript quantification task, with the restriction that transcript abundances must be consistent with the frequencies of dependent or independent processing events. Our algorithm runs in polynomial time even if a gene may generate an exponential number of isoforms, assuming the isoforms may be represented compactly as a PSG. Through experiments on real and simulated data, we assess the accuracy of the estimates from our method and demonstrate its theoretically predicted advantage over a more simplistic junction-read (JR) approach that does not make full use of the data. Our parameter estimation method forms the basis of a couple of DP tests, which we show to have low false-positive (FP) rates using real replicate data and superior true-positive (TP) rates on simulated data.

Second, we provide conditions under which a PSG is provably identifiable given RNA-Seq data, conditions which are less restrictive than those for full-length isoform models. Thus, the biologically motivated assumption of independence between processing events has an important statistical side benefit.

Lastly, we argue that transcriptome assemblers should report PSGs rather than sets of full-length isoforms, particularly when the data do not provide sufficient information to determine dependencies between processing events. The PSG framework offers an alternative parsimony objective in this context: that of minimizing the number of processing event parameters, rather than the number of isoforms, needed to explain the data. The fact that many transcriptome assemblers already build splice graphs as intermediate data structures makes PSGs a natural fit for these assemblers. To facilitate integration of our method with *de novo* assemblers, our method has been implemented in such a way that a reference genome is not required. Thus, our software may be used in conjunction with such assemblers to study alternative splicing in species without a sequenced genome.

### 1.1 Related work

One of the first descriptions of PSGs appears in Jenkins *et al.* (2006), which focuses on EST analysis. In contrast to our application of PSGs to short RNA-Seq data, parameter estimation for their models was trivial due to the use of only longer ESTs that mapped uniquely to full-length isoforms. Chang *et al.* (2005) also described the concept of a PSG, but did not use it in their EST-based methods. Xing *et al.* (2006) used splice graphs for EST analysis, but treated them simply as data structures with which to enumerate all possible full-length isoforms of a gene rather than as probabilistic models of isoform frequencies.

Non-probabilistic splice graphs have been used in the context of RNA-Seq as well. For example, Montgomery *et al.* (2010) summarized RNA-Seq data in terms of read coverage (‘flux’) of each edge in a splice graph and estimated abundances of known full-length transcripts using a non-statistical L1-minimization formulation. Singh *et al.* (2011) also computed observed read coverages for edges of a splice graph but instead used these values to detect DP between samples using non-parametric statistical tests. In contrast, our methods are based on generative probabilistic models of both RNA-Seq data and isoform frequencies with splice graph edges weighted by parameters representing RNA processing conditional probabilities. These models enable us to use more powerful parametric statistical techniques for both estimating isoform and processing event frequencies and detecting DP genes between samples.

For the task of gene annotation, Rogers *et al.* (2012) developed SpliceGrapher, which constructs splice graphs from RNA-Seq and EST data. Our methods assume that the splice graph structure is known and thus could work in tandem with SpliceGrapher to infer both the structure and parameters of PSGs.

The model of RNA-Seq read generation that we use is similar to the models assumed by many other methods for transcript quantification (Bohnert *et al.*, 2009; Jiang and Wong, 2009; Katz *et al.*, 2010; Li *et al.*, 2010a; Nicolae *et al.*, 2010; Richard *et al.*, 2010; Trapnell *et al.*, 2010). The goal of these methods is generally to estimate full-length isoform frequencies directly, and thus they suffer in the face of the challenges we have described. Others have focused on quantifying the frequencies of subcomponents of isoforms, such as individual exons (Kakaradov *et al.*, 2012; Katz *et al.*, 2010). In contrast to these methods, our approach allows for frequency estimates at all levels simultaneously, from individual processing events to the exon and full-length isoform levels.

### 1.2 Probabilistic splice graphs

PSG*s* are statistical extensions of *splice graphs*, data structures that can compactly represent all isoforms of a gene and show the structural relationships among them. The *splice graph* of a gene was originally defined by Heber *et al.* (2002) as a directed acyclic graph, 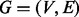, with a vertex for each exonic genomic position of the gene and an edge from vertex *v* to vertex *u* if the corresponding genomic position of *v* immediately precedes that of *u* in some isoform of the gene. Typically, one merges vertices *v* and *u* if 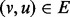 and 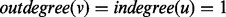. Thus, in general, the vertices of a splice graph represent exonic *segments* of a gene. The key property of a gene’s splice graph is that every isoform of the gene corresponds to a path through the graph.

In this article, we use a splice graph definition similar to that of Heber *et al.* (2002), but that differs in two respects. First, we allow a single genomic position to correspond to multiple vertices. For example, two vertices might represent the same exon with slightly different donor or acceptor sites. Second, we require that a splice graph has two additional vertices, representing the start and end of an isoform. The start vertex has edges to each possible transcription start site of a gene and the end vertex has incoming edges from each possible polyadenylation site. The start and end vertices are both associated with empty sequences (i.e. they do not represent exonic segments). Because of the inclusion of these special vertices, edges of a splice graph represent all classes of AP events.

Following the work of Chang *et al.* (2005) and Jenkins *et al.* (2006), we define a PSG as a weighted splice graph in which each edge is assigned a weight in [0, 1] and the weights of all edges out of a vertex sum to one. The edge weights in a PSG represent conditional probabilities of different AP events. The probability of an isoform is defined as the product of the weights of the edges along the path through the graph representing the isoform. Thus, a PSG represents the relative abundances of the possible isoforms of a gene. The principal assumption of a PSG is that an AP event at a given point along a path from the start vertex to the end vertex is independent of AP events that occurred before that point. PSGs are thus Markovian and model AP events as occurring in a 5′–3′ order, which is biologically motivated by the fact that splicing can occur co-transcriptionally (Dye *et al.*, 2006). The independence statements assumed by a PSG allow it to compactly specify the probabilities of all possible isoforms.

**Fig. 2.**
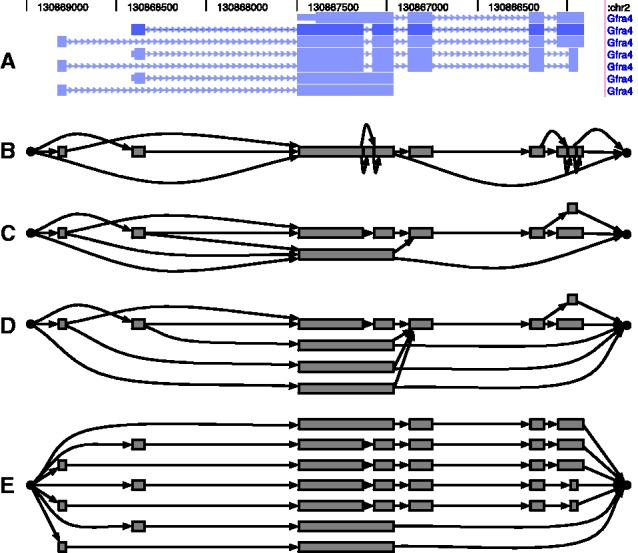
Example PSG representations for the mouse gene *Gfra4*. (**A**) A UCSC Genome Browser visualization of the seven annotated isoforms of this gene. (**B**) The line graph PSG. (**C**) The first-order exon graph. (**D**) A higher-order exon graph. In this graph, the AP events immediately following the longest exon are allowed to depend on the AP event directly preceding the exon, in contrast to the first-order exon graph, in which these AP events are independent of each other, given that the longest exon is included in the transcript. (**E**) An unfactorized PSG

Many PSG structures can be used to model the set of isoforms for a gene. For example, Figure 2 gives four PSGs that all represent the mouse gene *Gfra4*, which has seven possible isoforms according to the UCSC Genes annotation (Hsu *et al.*, 2006). These different PSGs are closely related to the various forms of splice graphs that have been used in splice graph databases (Bollina *et al.*, 2006). Like the splice graphs in these databases, PSGs can vary in the number of isoforms they allow. In addition, PSGs can vary in the family of probability distributions they define over the set of isoforms.

The PSGs of Figure 2B and C are examples of PSGs for the same gene that model different numbers of isoforms. Figure 2B is a specific type of PSG that we will refer to as a *line graph*, following Bollina *et al.* (2006). This type of PSG is equivalent to the ‘pairwise model’ considered by Jenkins *et al.* (2006). A line graph assumes independence between all compatible AP events.

The PSG of Figure 2C also belongs to a particular class of PSGs that we will refer to as *exon graphs*. In a *first order exon graph*, each exon is represented by a single vertex. In Bollina *et al.* (2006), such a graph is simply referred to as a ‘splicing graph’. Exon PSGs allow for dependencies between the AP events at the ends of each exon. Figure 2C and D are examples of PSGs that allow for the same set of isoform structures, but that differ in the families of distributions they define over the isoforms. Figure 2D is an example of a *higher order exon graph*, which generally has multiple vertices representing a single exon, and is analogous to a higher order Markov chain. In the most extreme case, a PSG can represent each possible isoform as a disjoint path through the graph (e.g. Fig. 2E). We refer to such PSGs as *unfactorized graphs*. Unfactorized graphs have structures equivalent to what are commonly displayed in genome browsers and are statistically equivalent to the full-length isoform models used by most previous quantification methods.

For the experiments we describe in Section 3, we will make use of line, exon and unfactorized graphs. For a given gene, the set of AP event dependencies that are known or that one wishes to allow during inference govern the complexity of the PSG that should be used. If one uses a less complex PSG model (e.g. a line graph) that happens not to model some true dependencies, the individual edge weights of the PSG may be accurate, but the full-length isoform frequencies implied by the PSG will likely be off.

## 2 METHODS

In this article, we assume that we are provided with a fixed PSG structure for a gene and focus on inference tasks given RNA-Seq data. PSG structures may be constructed using known gene annotations or inferred from RNA-Seq data by other methods. In this section, we describe methods for two tasks: (i) estimating the parameters of a PSG, and (ii) determining whether a gene or splice junction is differentially processed between two samples.

This section is structured as follows. We first formally define a PSG and a number of useful quantities computed from a PSG. We then describe a model of RNA-Seq data given a PSG. The identifiability of this model will then be addressed, followed by a description of how the EM algorithm is used to determine MAP parameters. We then provide simple likelihood ratio tests for detecting genes or splice junctions that are differentially processed between two samples. Our model, its associated inference methods and arguments about its identifiability are all independent of the specific form of PSG used (e.g. a line or unfactorized graph).

### 2.1 PSG notation and derived quantities

A PSG is a directed acyclic graph (DAG), 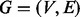, with a start vertex, *v*_0_, and an end vertex, *v_M_*, where 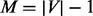. The only vertex in the graph with 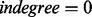 is *v*_0_ and the only vertex with 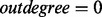 is *v_M_*. Each vertex, *v_i_*, of a PSG is associated with a sequence, which we denote by 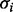. The sequences of the start and end vertices are the empty string. Each edge, 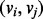, in the graph has a weight 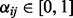, and we require that 
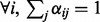
. The weight, *w*(*s*), of a subpath, *s*, is the product of the weights of its edges:

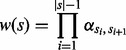



An isoform is represented by a path *t*, with 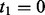 and 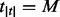. The probability of a transcript *t* is defined as the weight of its path, *w*(*t*).

Several useful quantities can be computed from a PSG that have not been described previously. These quantities will be important for specifying the RNA-Seq model and efficiently estimating parameters using the EM algorithm. Because a PSG is a DAG, each of these quantities can be described by a recurrence and computed efficiently using dynamic programming. First, we can compute the conditional probability that vertex *v_j_* is included in a transcript given that *v_i_* is in the transcript. We denote this quantity by 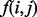 and compute it with the recurrence

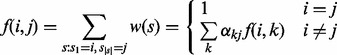

Other useful quantities involve the lengths of transcripts or subpaths. We denote by 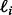 the length of the sequence associated with vertex *i*, i.e. 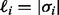. The length of a subpath *s* is simply the sum of the lengths of the sequences associated with its vertices: 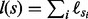. We define the expected prefix length 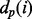 for vertex *v_i_* to be the expected length of the subpath beginning at *v*_0_ and ending at *v_i_*; analogously, the expected suffix length 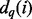 for vertex *v_i_* is the expected length of the subpath beginning at *v_i_* and ending at *v_M_*. These quantities can be calculated via the recurrences:
(1)
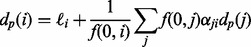

(2)
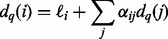

The expected length of transcript of this gene is the expected suffix length of *v*_0_ or the expected prefix length of *v_M_*, 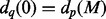.

### 2.2 A PSG RNA-Seq model

We now present a novel generative model for RNA-Seq data given a PSG, *G*, that describes the relative abundances of isoforms of a gene. This model will allow us to estimate the parameters of *G* given RNA-Seq data. Our model is a PSG generalization of the models that require lists of full-length isoforms (Li *et al.*, 2010a; Katz *et al.*, 2010; Trapnell *et al.*, 2010). With an unfactorized PSG, our model is equivalent to the full-length isoform models.

To simplify our presentation, we will describe a model of single-end reads without sequencing error. We provide the details of model extensions for paired-end data and sequencing error in the Supplementary Material, and our software accommodates both of these issues, including the handling of reads that align to multiple positions. The Supplementary Material also describes how to efficiently simulate data from our model.

We assume that an RNA-Seq dataset represents *N* fragments, each independently derived from one of the possible isoforms allowed by *G*. The RNA-Seq data consist of reads from one end of each of the *N* fragments, each read of length *L*. The single-end model involves four random variables for each of the *N* reads: *R_n_*, the sequence of read *n*; *T_n_*, the full transcript path from which read *n* was derived; *S_n_*, the subpath of *T_n_* from which read *n* is derived; and *B_n_*, the position in the sequence of 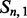 at which read *n* begins. Of these random variables, only *R_n_* is observed. In addition, the latent PSG edge weights 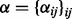 are also treated as random variables. The joint probability distribution over the random variables is

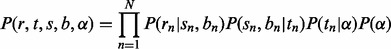

Assuming no sequencing error, we have that

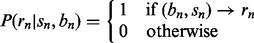

where 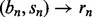 denotes that *r_n_* is the length *L* sequence starting at position *b_n_* in the concatenation of sequences 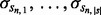. If 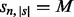, then the concatenated sequence also includes an infinitely long sequence of As, representing the poly(A) tail at the end of a typical eukaryotic protein-coding transcript. We will often use the notation 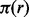 to refer to the set 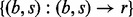. In addition, we say that *r* is *derived* from *s* if there exists some *b* such that 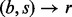.

We assume that the position *b_n_*, at which a read begins, is uniformly distributed across the length of the transcript from which it is derived. Thus,

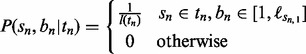



We assume that the probability of generating a read from a specific transcript, *t_n_*, is proportional to the product of the relative frequency of the transcript, 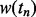, and the length of the transcript:

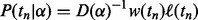

where 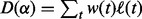, which is the expected length of a transcript given the PSG. Finally, for each vertex *i*, we place a Dirichlet prior with parameters 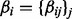 on the weights of its out-edges. Specifically,

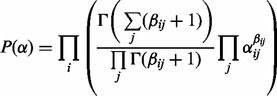



### 2.3 Identifiability of the PSG RNA-Seq model

A PSG RNA-Seq model is more likely to be identifiable than a model over all full-length isoforms implied by the PSG. In the Supplementary Material, we state some general conditions under which a PSG is guaranteed to be identifiable. These conditions are generalizations of those for the identifiability of full-length isoform models (Hiller *et al.*, 2009; Lacroix *et al.*, 2008). Here we provide a simple set of specific conditions that are sufficient, but not necessary, for the identifiability of a PSG.

**Proposition 1**

*If for each edge* 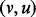 *in a PSG at least one of the following conditions is true, then the model is identifiable*.

1. *There is a read that is uniquely derived from* 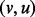
*.*
2. *There is a read that is uniquely derived from u and* 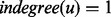.

A proof of this proposition is provided in the Supplementary Material. It provides an easy check for whether a PSG is identifiable: simply determine if each edge or its target vertex can produce a unique read. These criteria are generally easier to satisfy than those required for the identifiability of full-length isoform models. For example, the PSG in Figure 1E is identifiable, even though a model of the full-length isoform frequencies is not.

### 2.4 Edge weight estimation using EM

Given a set of aligned RNA-Seq data, we compute MAP estimates of the PSG edge weights. When 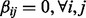, this is equivalent to computing maximum likelihood (ML) estimates for the α values (treated as parameters). We use the EM algorithm to compute these estimates, as the probability 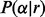 is difficult to optimize directly. Briefly, the E-step of the EM algorithm involves computing the expected number of times each edge of the PSG is used in a transcript from which a read is derived. The M-step then sets α to maximize the joint probability given these expected counts. The full details of the application of the EM algorithm to our model are provided in the supplement. One detail of our algorithm that is critical to its efficiency is our extensive use of the 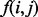, 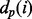 and 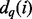 values, which are computed via dynamic programming. With these values precomputed during each iteration of the E and M steps, these steps run in 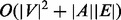 and 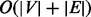 time, respectively, where *A* is the set of alignments of all reads. Similarly, the memory required by the algorithm is 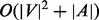. Without dynamic programming, the EM algorithm would require time and memory proportional to the number of possible full-length isoforms, which may be exponential in 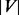.

### 2.5 Testing for DP

To test for DP of a gene between two samples we use a simple likelihood ratio test. Given two read sets, *R*^1^ and *R*^2^, we compute the ML parameters, 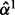 and 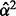, for the two sets separately, as well as the ML parameters, 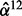, for the two sets combined. We test the null hypothesis that the parameters for the two samples are the same by computing the ratio

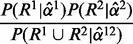

and assigning a *P*-value using a 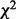 distribution with *k* degrees of freedom, where *k* is the number of free parameters in the PSG. When predicting DP genes across an entire genome, a *P*-value significance threshold is selected according to the Benjamini–Hochberg procedure for controlling the false discovery rate.

We can additionally test for DP of an individual splice site or the transcription start site using a similar technique. We select a single vertex, *v*, and test the null hypothesis that the parameters for the out-edges of that vertex are identical between the two samples with all other edge weights possibly different. The alternative hypothesis is the same as in the gene-level test. After estimating ML parameters for these two hypotheses, we perform a likelihood ratio test with 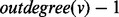 degrees of freedom.

### 2.6 Software

Our methods are implemented in a freely available software package called PSGInfer. The software currently takes as input FASTA-formatted genomic sequences, GTF-formatted transcript annotations and FASTQ-formatted RNA-Seq data. PSGs may be provided directly in lieu of a reference genome and annotation when, for example, a *de novo* transcriptome assembly is used. For single samples, the software outputs estimates of the frequencies of processing events and annotated full-length isoforms. For pairs of samples, it outputs the results of the DP test on each gene.

PSGInfer comprises three major scripts, psg_prepare_reference, psg_infer_frequencies and psg_infer_diff_processing. A typical workflow for using these scripts is depicted in Supplementary Figure S1. The psg_prepare_reference script is responsible for constructing splice graphs from a set of known transcript annotations. It can be configured to produce line, exon or higher-order PSGs, such as depicted in Figure 2. The psg_infer_frequencies script aligns a single RNA-Seq sample against a prepared set of splice graphs using Bowtie (Langmead *et al.*, 2009) and computes frequency estimates. Lastly, the psg_infer_diff_processing script takes the output of psg_infer_frequencies from two samples and reports DP predictions.

## 3 RESULTS

We performed a variety of experiments on both simulated and real RNA-Seq data to analyze the accuracy and performance of our PSG methods. On simulated data, we first quantify the theoretical advantage of our method over a more simplistic JR–based method for estimating processing event frequencies. On real *Drosophila* data, we compare the estimates from the PSG and JR method and measure the improvement in the convergence rates of our method’s estimates with bootstrapping experiments. We then evaluate the accuracy of our DP tests through experiments on sets of real and simulated RNA-Seq samples that include biological and technical replicates. Lastly, with real data from the *Drosophila* gene *Dscam*, we demonstrate the superior time complexity of using a factorized splice graph-based method.

### 3.1 Comparison with a JR approach

The EM-based method we have described for estimating the parameters of a PSG draws power from every alignable read in an RNA-Seq dataset. That is, in our EM method, every read is potentially informative about the frequencies of all AP events. However, there are other more simplistic methods that may be used to estimate the parameters of a PSG. In general, these methods ignore some subset of the data and are thus less powerful than the EM method when the model assumptions hold.

One such approach is to consider only those reads that align across edges (junctions) in the splice graph and to compute MAP estimates of the edge parameters using the number of reads that map to each edge. We will refer to this more simplistic strategy as the JR approach. Although this method is simplistic and ignores much of the data, it is statistically consistent under our RNA-Seq model, with JR estimates converging to true parameter values as the sample size becomes large. In addition, it is relatively robust to violations of the assumptions made in our RNA-Seq model, such as the assumption that the probability of a read being generated by a transcript is proportional to that transcript’s length. And while it is sensitive to non-uniformities around individual junctions, it is not affected by biases in the read distributions elsewhere in the transcripts and the estimates at one junction do not affect those at other more distant junctions.

An article published during the revisions of this manuscript demonstrated that a JR approach can provide more accurate estimates than more sophisticated methods that use reads mapping to exon bodies (Kakaradov *et al.*, 2012). For example, Katz *et al.* (2010) describe a method (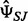) that uses reads mapping to exon bodies immediately flanking a junction, in addition to reads mapping to the junction itself. As this method is only appropriate for certain types of alternative splicing events (e.g. cassette exon inclusion), we do not consider it here. In addition, we expect that the 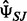 method would provide comparable estimates to that of our EM method for AP events such as cassette exons, as the two methods make similar use of the data in these situations. Therefore, we focus on comparisons with the JR method, which can provide estimates for all AP events, with the exception of alternative transcription start site events, which do not involve a junction.

#### 3.1.1 Comparisons on simulated data

We first sought to quantify the theoretical advantage of our EM method over that of the JR approach using data simulated according to our model, as was similarly done in Katz *et al.* (2010). Of course, these experiments represent the best-case scenario for the EM method, as the data perfectly fit the model that the EM method uses to gain power. Nevertheless, these experiments allow us to quantify the size of the improvement in the accuracy of the EM estimates over those of JR when the model assumptions hold.

For our simulations, we used protein-coding *Drosophila* genes from the FlyBase v5.12 (Smith *et al.*, 2007) annotation, each containing a single cassette exon. To capture a range of scenarios, we ordered all such genes by the lengths of their cassette exons and selected the three genes at the quartiles of this distribution. We simulated single-end reads of length 100 from the genes, each of which can be represented generically by the gene model shown in Supplementary Figure S2A. JR and EM were used to estimate the one parameter of the line PSG for this gene on simulated read sets varying in size from 10 to 10 000 reads, with 500 simulations per read set size. For both methods, we used a pseudocount of one (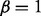 for EM) for the splice junctions, which helps to control the variance of the estimates on small datasets. As expected, owing to the fact that EM extracts more information from the data than JR, the EM estimates converge more quickly to the truth and exhibit less variance than those of JR (Supplementary Fig. S2B). The advantage of EM over JR is most pronounced for read set sizes 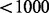 and for the gene with the longest cassette exon (*s-cup*), as EM gains information from reads that map to the body of that exon. Simulations with paired-end reads gave similar results, with both methods benefitting from paired-end data (Supplementary Fig. S3).

#### 3.1.2 Comparisons on real data

We next compared the estimates of the EM and JR methods on a real set of *Drosophila* RNA-Seq data to assess the impact of the model assumptions used by EM. Here we assumed that the more robust JR estimates are accurate when a large number of reads are available. We used RNA-Seq data from the *Drosophila* cell line CME-W1-Cl.8+ (SRA accession SRS002587) (Cherbas *et al.*, 2011), which consists of 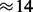 million pairs of reads, each 37 bases long. Gene annotations were obtained by selecting all protein-coding genes of FlyBase v5.12 and preprocessing using Cufflink’s cuffcompare program. We used Bowtie (Langmead *et al.*, 2009) to align the reads against the sequences of the line PSGs constructed from these annotations and selected all 88 genes that (i) included at least one AP event and (ii) had more than 5000 read pairs mapped to it.

**Fig. 3.**
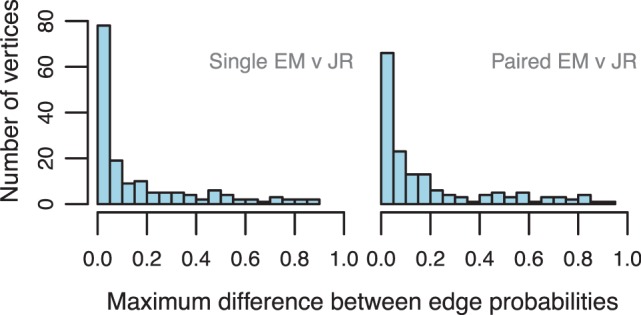
Distributions of the differences between the parameter estimates of EM and JR from single and paired-end data

The line PSG parameters for each of these genes were estimated using JR and EM. For each vertex with outdegree 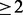, we computed the distance between the probabilities of its out-edges by taking the maximum of the absolute difference between the estimates on each edge (infinity norm). Figure 3 gives the distributions of these distances between EM and JR estimates for both single and paired-end reads (Supplementary Fig. S4 gives the plots for comparisons between estimates from the same method on single and paired-end reads). We also examined how often the estimates at each vertex agreed in terms of which AP event following that vertex was most likely. EM and JR agreed with respect to this measure on 84 and 81% of the vertices for single and paired-end estimates, respectively. The single-end and paired-end estimates agreed with each other on 95 and 93% of vertices for EM and JR, respectively. These results indicate that the EM estimates are highly accurate on average, assuming that the JR estimates are close to the truth. This suggests that the model assumptions used by EM are reasonable, at least on this dataset. The differences observed between the estimates of the same method on single and paired-end data show that many of the discrepancies between the methods arise simply because they draw information from different subsets of the data. The remaining discrepancies may be the result of highly biased read distributions or incorrectly annotated gene structures.

We further explored the validity of the independence assumptions made by the line PSGs in these experiments by additionally computing estimates of the AP event frequencies with first-order exon and unfactorized PSGs for these genes. As is shown in Supplementary Figure S5, the estimates from the line, first-order exon and unfactorized PSGs are all similar in terms of their distances from the reference JR estimates. Thus, the independence assumptions made by the line PSGs do not appear to affect the accuracy of the AP frequency estimates for this dataset. This experiment also shows that AP frequency estimates from factorized PSGs are comparable with those estimated from full-length isoform models, as the unfactorized PSGs are equivalent to such models.

**Fig. 4.**
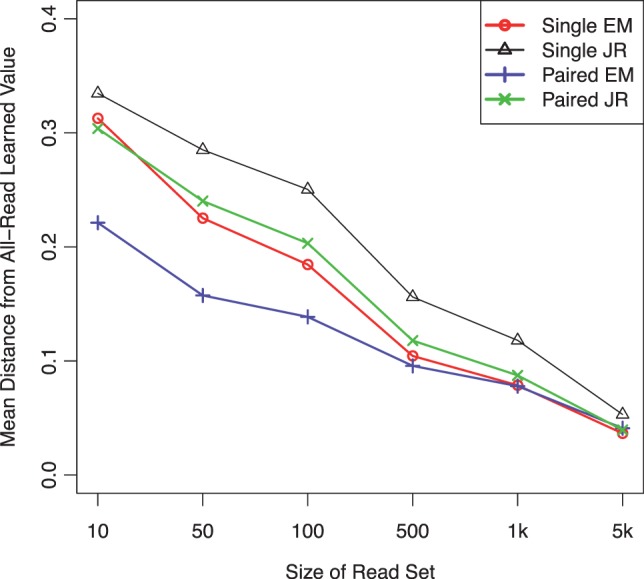
The mean distances of parameter estimates on bootstrap samples from those on the full read set as a function of the bootstrapped read sample size. All differences between pairs of methods at each read set size are significant (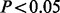, sign test) except for that between Single EM and Paired JR for read set size = 10

We additionally assessed the convergence rates of JR and EM through bootstrapping experiments on these data. For each of the selected 88 genes, we generated a series of bootstrapped read samples from its full read set. We generated read samples of sizes ranging from 10 to 5000, with 100 samples per read set size. On each read sample we estimated the line PSG parameters with both JR and EM and calculated the distance between each method’s estimates and its estimates on the full read set. Mean distances were computed for each read set size and a further mean was taken over all vertices (Fig. 4). As expected, the distances approach zero as the read set size increases, EM estimates converge faster than those of JR and estimates from paired-end data converge the fastest. EM with single-end data converges about as quickly as JR with paired-end data, suggesting that for this dataset, EM is extracting about twice as much information from the data as JR.

### 3.2 Evaluation of DP calls

We evaluated the performance of our PSG-based DP test by applying it genome-wide on four sets of RNA-Seq samples: three real and one simulated. The four sets were five modENCODE *Drosophila* samples (Cherbas *et al.*, 2011), four human samples from HapMap individuals (Montgomery *et al.*, 2010), four human cell line samples from the ENCODE project (The ENCODE Project Consortium, 2011) and four samples simulated using parameters learned from the ENCODE human samples. The real sets were selected because they were publicly available, contained paired-end data, which benefit DP analyses and contained either biological or technical replicates. Beyond these criteria, the sets we have presented were chosen arbitrarily and *a priori*. Details of these sets are provided in the supplement.

Using first-order exon PSGs, we applied our DP test to all pairs of samples within each set to assess its FP and TP rates. We expected that an accurate DP test should call few genes as DP between replicates and a modest number of genes as DP between non-replicate samples. For comparison, we also applied FDM (Singh *et al.*, 2011) and Cuffdiff 2 (Trapnell *et al.*, 2013) to these datasets. The parameters and annotations used to run the PSG, FDM and Cuffdiff DP tests are described in the supplement. The target false discovery rate, as determined by the Benjamini–Hochberg procedure, was set to 0.05 for all methods. We tallied the number of genes called as DP between each pair of samples for each method individually as well as the number of genes called as DP by multiple methods. Lists of the genes called as DP are provided on our software’s Web site.

Table 1 summarizes the results for the DP analyses on the three real datasets. Remarkably, the PSG test calls few genes as DP between the biological and technical replicates in the fly and HapMap datasets, indicating a low FP rate on these sets. At the same time, the test calls a modest number of genes as DP between non-replicate samples in these sets, suggesting that its low positive rate does not come at the expense of predicting TPs. On the ENCODE set, the number of DP calls between the replicate samples is also lower than that between the non-replicates. However, the FP rate is much higher for this set, particularly for the K562 replicates. This suggests that the ENCODE samples are more heterogeneous, perhaps owing to higher biological or sample preparation variability. One difference between the ENCODE samples and the others is that the read length is more than twice as long in the ENCODE samples. However, through an experiment in which we reran the DP tests with the ENCODE reads trimmed to match the read lengths in the other sets, we did not find this to be the cause of the larger numbers of PSG DP calls for these samples (Supplementary Table S1).

**Table 1.** The number of DP genes called by the PSG test, FDM, Cuffdiff and combinations of the methods on pairs of samples from three sets: (A) HapMap, (B) *Drosophila* modENCODE and (C) ENCODE

Sample 1	Sample 2	PSG	FDM	Cuffdiff	PSG 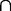 FDM	PSG 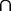 Cuffdiff	FDM 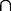 Cuffdiff	All
(A)								
**CEU Rep 1**	**CEU Rep 2**	**0**	**0**	**1187**	**0**	**0**	**0**	**0**
CEU Rep 1	Yoruban Rep 1	39	24	269	2	8	3	1
CEU Rep 1	Yoruban Rep 2	46	24	282	3	5	3	1
CEU Rep 2	Yoruban Rep 1	45	22	253	4	5	1	1
CEU Rep 2	Yoruban Rep 2	38	29	260	2	4	4	1
**Yoruban Rep 1**	**Yoruban Rep 2**	**0**	**0**	**1253**	**0**	**0**	**0**	**0**

(B)								
**CME_W1_Cl.8+ Rep 1**	**CME_W1_Cl.8+ Rep 2**	**16**	**32**	**204**	**1**	**0**	**2**	**0**
CME_W1_Cl.8+ Rep 1	Kc167	365	207	7	75	2	0	0
CME_W1_Cl.8+ Rep 1	ML-DmBG3-c2	232	164	6	46	1	1	0
CME_W1_Cl.8+ Rep 1	S2-DRSC	406	228	12	86	6	1	1
CME_W1_Cl.8+ Rep 2	Kc167	319	211	16	72	4	3	1
CME_W1_Cl.8+ Rep 2	ML-DmBG3-c2	260	126	16	37	2	1	1
CME_W1_Cl.8+ Rep 2	S2-DRSC	353	220	17	71	5	1	1
Kc167	ML-DmBG3-c2	384	321	12	93	2	1	1
Kc167	S2-DRSC	419	209	12	88	6	2	1
ML-DmBG3-c2	S2-DRSC	431	287	4	110	3	1	1

(C)								
**HUVEC Rep 1**	**HUVEC Rep 2**	**35**	**43**	**440**	**6**	**2**	**4**	**1**
HUVEC Rep 1	K562 Rep 1	376	344	8	113	2	0	0
HUVEC Rep 1	K562 Rep 2	379	302	12	81	6	2	2
HUVEC Rep 2	K562 Rep 1	442	382	8	144	4	3	3
HUVEC Rep 2	K562 Rep 2	355	285	10	80	3	2	2
**K562 Rep 1**	**K562 Rep 2**	**224**	**308**	**168**	**39**	**8**	**8**	**1**

*Note*: Pairs of samples that are technical or biological replicates are indicated in bold.

The FDM method performed similarly to our PSG DP method, generally reporting fewer DP genes between replicates than between non-replicates. However, for all sample sets in Table 1, the PSG method reports more DP genes between non-replicate samples than FDM, while at the same time reporting smaller numbers of DP genes between replicate samples than FDM (except for in the HapMap set where both methods report zero DP genes between replicates). This result suggests that the PSG test has a higher TP rate and a lower FP rate than FDM. Surprisingly, of the genes called DP by either PSG or FDM, only a small fraction are called by both methods. The fraction shared is smallest for the replicate pairs of samples, which is expected given that all of the DP calls on these pairs are FPs. We hypothesize that the small overlap of the DP calls on the non-replicate pairs is owing to the fact that the two methods are different: our PSG DP method is a parametric test based on a generative model of the read data, whereas FDM is a non-parametric test that acts on read coverage levels. Also of note are the numbers of FDM DP genes for the trimmed ENCODE set (Supplementary Table S1), which are much smaller than those for the untrimmed set. We believe this occurs because the trimming of reads reduces read coverage levels, which are used by FDM.

Cuffdiff, on the other hand, had odd behavior in these experiments, as it called large numbers of genes as DP between replicates and a small number as DP between non-replicates. After discussing these results with the authors of Cuffdiff, we believe that they are explained by the combination of Cuffdiff accurately estimating low global processing variability between the replicate samples but then having highly noisy estimates of isoform frequencies for a subset of genes, particularly those that have multiple highly similar isoforms. When Cuffdiff is given a pair of non-replicate samples, it estimates higher levels of global variability and thus the effect of noisy isoform frequency estimates is diminished. This explanation fits with the general trend observed in the results in which the lower the variability between the samples, the larger the number of genes predicted as DP by Cuffdiff. Although Cuffdiff was originally used in an experiment without replicates (Trapnell *et al.*, 2010), during the revisions of this article, the authors of Cuffdiff began advising its users through its Web site to not use its DP tests with fewer than three replicates per condition, likely owing to issues such as the one we have observed here.

The performance of the three methods on the simulated dataset was similar to that on the real datasets (Supplementary Table S2). In this dataset, four samples were simulated with two biological replicates for each of two conditions (A and B). Each sample was simulated with separate parameter settings estimated from one of the four real human ENCODE samples (e.g. the simulation parameters for A Rep 1 were estimated from HUVEC Rep 1). Within replicates of the same condition, the relative isoform frequencies were set to be identical. We note that the gene-level abundances were different across all samples, even if the relative isoform frequencies were the same. Between conditions, 10% of multi-isoform genes expressed in both conditions were set to be DP, with isoform frequencies randomly shuffled between the two conditions for these genes.

Because we knew the true set of DP genes between the two conditions, we were able to measure the recall and precision of the methods on pairs of samples from different conditions, in addition to the number of FPs measured on pairs of samples from the same condition. The PSG DP method exhibited good FDR control with a precision of 0.93–0.95 and had the highest recalls (0.54–0.60) of the three methods. Although Cuffdiff displayed the same high FP behavior between pairs of replicate samples, it had reasonable precision (0.88–0.98) between non-replicate samples but with lower recall (0.11–0.13). FDM had moderate recall (0.24–0.39) but poor precision (0.40–0.51). Precision-recall curves constructed by varying the *P*-value threshold required for calling a gene as DP further demonstrated that the PSG method’s performance dominated that of FDM and Cuffdiff on this simulated dataset (Supplementary Fig. S6).

Because Cuffdiff and FDM are able to take into account multiple biological replicates per condition, we additionally performed an A versus B DP test in which all replicates were provided to the methods at the same time. Cuffdiff’s recall improved noticeably with this test, although with a slight decrease in precision (Supplementary Table S3). FDM, on the other hand, did not predict any DP genes with this test, which was the result of the highly conservative permutation test it uses when multiple replicates are provided. However, in terms of its precision-recall curve performance, FDM improved slightly at the high precision end of the curve, as compared with its performance with no replication (Supplementary Fig. S7). Despite only taking into account pairs of samples, the average accuracy of the PSG method between non-replicate pairs was superior to the accuracy of both Cuffdiff and FDM when these methods were provided with multiple biological replicates (Supplementary Table S3 and Supplementary Fig. S7).

One implementation detail that sets our PSG methods apart from Cuffdiff and FDM is that our PSG methods align RNA-Seq reads to transcript subsequences, whereas Cuffdiff and FDM accept genomic alignments. Although we used standard alignment options for our genomic alignments using TopHat (Trapnell *et al.*, 2009), we observed that the TopHat alignments generally had fewer aligned reads than the transcript alignments used by our PSG methods. To test if this factored into the performance differences on the simulated data, we additionally constructed a set of genomic alignments by aligning first to the annotated transcript sequences and then transforming to genomic coordinates. Running Cuffdiff and FDM with these alignments somewhat decreased FPs on the replicate pair tests, but did not markedly change recall and precision on the non-replicate pair tests with a target FDR of 0.05 (Supplementary Table S2 and Supplementary Fig. S6). Even with more comparable alignment inputs, the PSG method outperformed FDM and Cuffdiff on these data.

### 3.3 Efficiency of computation with an exon graph PSG

One advantage of the PSG approach is that inference can be more efficient, particularly for genes with many isoforms. Even if a gene has an exponential number (in terms of the number of exonic segments) of isoforms, a compact PSG model of the gene can enable polynomial-time inference of the model parameters. Although factorized PSGs only indirectly model the abundances of individual isoforms, they enable an efficient alternative for quantification.

To demonstrate these theoretical efficiency gains, we attempted to perform inference with the *Drosophila* gene *Dscam*, which is capable of producing tens of thousands of isoforms (Schmucker *et al.*, 2000). For this experiment, we used all read pairs (184) that mapped to *Dscam* from the CME_W1_Cl.8+ Rep 1 sample analyzed in the previous section. Estimation of the 74 free parameters of the first-order exon graph PSG from this dataset required less than 3 s of computation. We then constructed a reference annotation set of all 23 976 possible isoforms (equivalent to the unfactorized version of the exon graph PSG) of *Dscam* for use with the quantification-only mode of Cufflinks (Trapnell *et al.*, 2010). A run of Cufflinks on this dataset required >90 GB of RAM and did not complete within 6 h. This experiment indicates that as the number of annotated splice variants for each gene grows, quantification methods that explicitly model the abundance of each isoform will not be computationally feasible. We note that with current gene annotation sets, which are not complete in terms of representing all possible isoforms for each gene, Cufflinks and our PSG method require a comparable amount of time. For example, with the simulated human dataset and comparable alignments from the previous section, both the PSG and Cufflinks methods required 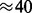 min per pair of samples (the PSG method additionally required 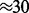 min of preprocessing time per individual sample) on a 12-core, 800 Mhz Linux server.

## 4 DISCUSSION

In this article, we have demonstrated the utility of PSG RNA-Seq models for analyzing AP of gene transcripts. Whereas splice graphs provide efficient *structural* representations of the possible isoforms of a gene, PSGs allow for compact *statistical* models of the relative frequencies of these isoforms. Because of their factorized nature, PSG models are more likely to be identifiable than full-length isoform models and we have provided some simple criteria wherein this is the case. In addition to being more identifiable, PSG models allow for efficient inference of event frequencies, even for genes with an exponential number of potential isoforms. Through our computational experiments with simulated and real data, we have confirmed that the event frequency estimates of the PSG model are accurate and converge more quickly than those of simpler JR approaches, as is theoretically expected. Lastly, we have shown that our DP tests achieve remarkably low FP rates on several real datasets and superior accuracy on simulated data.

Although the results of our DP experiments are promising for PSG-based tests, the lack of overlap between the predictions of the three compared methods is somewhat alarming. For example, on the fly non-replicate sample pairs, on average, only 21% of PSG DP predictions are shared with FDM. Our DP simulation experiment suggests that much of this lack of overlap is because of poor precision and recall by the other methods. However, large methodological differences may also be a factor here.

A limitation of our PSG-based DP test is that it is only designed to detect differences in the AP event frequencies between two samples and is unable to determine whether any such differences are biologically significant. This fact may explain our test’s poor performance on the K562 samples, which are likely to be highly variable. To determine if DP-called genes are biologically significant, one needs to use multiple biological replicates per condition (Hansen *et al.*, 2011). Currently, unlike FDM and Cuffdiff, our method cannot use replicates to assess biological variability. In addition, our methods do not currently model biases in RNA-Seq data, and therefore they are not suitable for comparing datasets generated by different protocols. Nevertheless, our method’s strong performance in distinguishing between replicate and non-replicate samples in the fly and HapMap datasets suggests that it is a good base method to be extended with models of biological variation and protocol bias. In addition, our simulations suggest that our PSG method can produce more accurate DP predictions than those from Cuffdiff and FDM, even when these methods are provided with multiple replicates per condition.

A major task that we have not addressed in this work is that of determining the best PSG structure for a gene. This task has two components: (i) identifying the exonic sequences, splice junctions, transcription start sites and polyadenylation sites of a gene, and (ii) determining the statistical dependencies between AP events. The first task is currently an active area of RNA-Seq research involving splice junction detection methods [e.g. Trapnell *et al.* (2009)], and reference-based (Guttman *et al.*, 2010; Rogers *et al.*, 2012; Trapnell *et al.*, 2010) and *de novo* transcriptome assembly (Grabherr *et al.*, 2011; Robertson *et al.*, 2010). The latter task can be thought of as a model selection problem, for which the statistical machinery we have presented should be invaluable.

In addition to the learning of PSG structures, our future work includes several extensions to our current model. First, we plan to modify the model to include sequencing biases, which have been demonstrated to significantly impact quantification from RNA-Seq data (Hansen *et al.*, 2010; Li *et al.*, 2010b; Roberts *et al.*, 2011). Second, we plan to implement a hierarchical Bayesian version of our model to properly handle the detection of DP with multiple biological replicates.

## Supplementary Material

Supplementary Data
