# Supplementary material for: Inference of alternative splicing from RNA-Seq data with probabilistic splice graphs
Source: Bioinformatics. 2013 Jul 11;29(18):2300–10. doi: 10.1093/bioinformatics/btt396 (PMC3753571; doi:10.1093/bioinformatics/btt396)
Supplement: Supplementary Data [file supp_29_18_2300__index.html]

Inference of alternative splicing from RNA-Seq data with probabilistic splice graphs — Inference of alternative splicing from RNA-Seq data with probabilistic splice graphs — Inference of alternative splicing from RNA-Seq data with probabilistic splice graphs — Supplementary Data 

# Inference of alternative splicing from RNA-Seq data with probabilistic splice graphs

## 

files

**Files in this Data Supplement:**

- Supplementary Data - pdf file
