# Supplementary material for: Inference of alternative splicing from RNA-Seq data with probabilistic splice graphs
Source: Bioinformatics. 2013 Jul 11;29(18):2300–10. doi: 10.1093/bioinformatics/btt396 (PMC3753571; doi:10.1093/bioinformatics/btt396)
Supplement: Supplementary Data [file supp_btt396_supplement.pdf]

Supplementary Material for "Inference of alternative splicing from RNA-Seq data with probabilistic splice graphs"

Laura H. LeGault and Colin N. Dewey

Contents

1 Model extensions **1**
1.1 Extension to paired-end reads 1
1.2 Extension for sequencing error 2

2 Efficient simulation with the PSG RNA-Seq model **2**
2.1 Single-end read generation 3
2.2 Paired-end read generation 3

3 Identifiability of PSG RNA-Seq models **3**

4 Computing MAP estimates of edge weights with EM **5**
4.1 E-step 6
4.1.1 Handling sequencing error and paired-end data 6
4.2 M-step 7

5 Data sets and methods for differential processing experiments **8**
5.1 DP tests on real data 8
5.2 DP tests on simulated data 10
5.3 Testing the impact of the RNA-Seq read alignments on DP accuracy . 11

6 Supplementary tables **12**

7 Supplementary figures **15**

1 Model extensions

1.1 Extension to paired-end reads

To extend our model to paired-end reads, we add an additional latent random variable, F_n, for each read that represents the length of fragment n, and take R_n to represent the pair of reads from the ends of the fragment. The S_n variable now becomes the subpath

of T_n from which the *fragment* is derived and B_n is the leftmost position at which the fragment starts in $S_{n,1}$. The joint probability with paired-end data is thus:

$$P(r,t,f,s,b,\alpha) = \prod_{n=1}^{N} P(r_n|f_n,s_n,b_n)P(s_n,b_n|t_n,f_n)P(f_n)P(t_n|\alpha)P(\alpha)$$

with $P(f_n)$ assumed to be normal distribution with specified mean and standard deviation and the other condition distributions modified accordingly. In our software, we require that the user provide the mean and standard deviation of the fragment length distribution.

1.2 Extension for sequencing error

We assume that each read is associated with a quality score string that provides estimates from the sequencer of the probability of an error at each position within the read. We denote the quality score string for read n by the random variable Q_n. The generation of the quality scores are not modeled, and thus we concern ourselves only with the conditional probability $P(r,t,f,s,b,\alpha|q)$. The only factor in this probability that is affected by the quality scores is that for the read sequences. The updated conditional probability for a read sequence is

$$\begin{aligned} P(r_n|s_n,b_n,q_n) &= \prod_i P(r_{n,i}|s_n,b_n,q_{n,i}) \\ &= \prod_i \epsilon(r_{n,i},q_{n,i},s_{n,b_n+i}) \end{aligned}$$

where ϵ is defined as

$$\epsilon(c_r,q_r,c_s) = \begin{cases} 1-10^{\frac{-q_r}{10}}, & c_r = c_s \\ \frac{1}{3}10^{\frac{-q_r}{10}}, & c_r \neq c_s \end{cases}$$

which follows from the probabilistic meaning of Phred quality scores.

2 Efficient simulation with the PSG RNA-Seq model

Simulating data from the model is straightforward given the model description in the main text. However, when the number of possible isoforms is large, simulation can be done more efficiently by taking advantage of the fact that

$$P(s,b) = D(\alpha)^{-1}f(0,s_1)w(s)$$

which allows one to avoid explicitly sampling a specific transcript.

2.1 Single-end read generation

To generate single-end reads from our PSG RNA-Seq model, we first calculate the probability that read n begins in vertex i:

$$
\begin{aligned}
P(S_{n,1} = i) &= \sum_t \sum_{j<\ell_i} P(S_{n,1} = i, B_n = j, T_n = t) \\
&= \sum_t \sum_{j<\ell_i} P(S_{n,1} = i, B_n = j | T_n = t) P(T_n = t) \\
&= \sum_{t:i\in t} \ell_i \frac{1}{\ell(t)} D(\alpha)^{-1} w(t) \ell(t) \\
&= D(\alpha)^{-1} \ell_i \sum_{t:i\in t} w(t) \\
&= D(\alpha)^{-1} \ell_i f(0, i)
\end{aligned}
$$

We select the beginning vertex according to this probability and select a starting position b uniformly within the sequence of this vertex. If the read does not fit entirely within the vertex, subsequent vertices in the read are selected according to edge probabilities α_{ij}. If the read runs off the end of the PSG, a poly(A) tail is added to the end of the read. Generating reads in this manner is more efficient than sampling a full path transcript and then choosing reads from the transcript, as the number of transcripts may be exponential depending on the complexity of the splice graph structure.

2.2 Paired-end read generation

The process for generating paired-end reads is similar to that for single-end reads. A fragment length is first sampled from a normal distribution and a fragment of that length is generated using the techniques of single-end read generation. A read of the specified read length is then generated from each end of the fragment.

3 Identifiability of PSG RNA-Seq models

In this section we prove that under some general criteria, a PSG RNA-Seq model is guaranteed to be identifiable. We first define some terminology necessary for understanding these criteria.

We say that vertex v *dominates* vertex u ($v \gg u$) if all paths from the start vertex to u include v. A vertex v *dominates* a read r ($v \gg r$) if for all alignments, (b, s), of r, $v \gg s_1$. For a vertex v with n children, $u_1, \ldots, u_n$, and a read r with $v \gg r$, the *dominated-read vector*, $\mathbf{x}^v$, for v and r is a length n vector with

$$
x_i^v = \sum_{(b,s)\in\pi(r)} \begin{cases} w(s) & s_1 = u_j \\ w(s_{2,\ldots,|s|}) & s_{1,2} = (v, u_j) \\ 1 & s = (v) \\ 0 & u_j \notin s \end{cases}
$$

A key property of the dominated-read vector for v_k and r is that

$$P(r|\alpha) = D(\alpha)^{-1} f(0,k)(\mathbf{x}^{v_k} \cdot \boldsymbol{\alpha}_k),$$

assuming that all reads (fragments) are of fixed length. For a vertex v with n children, $u_1, \ldots, u_n$, and m dominated reads, $r_1, \ldots, r_m$, the *dominated-read matrix*, M^v, for v is a $m \times n$ matrix with the ith row equal to the dominated-read vector for v and r_i. It follows that

$$D(\alpha)^{-1} f(0,k) M^{v_k} \boldsymbol{\alpha}_k = \mathbf{P}^{v_k}$$

where $\mathbf{P}^{v_k}$ is a vector with ith entry $P(r_i|\alpha)$.

Proposition 1. *For a PSG RNA-Seq model with fixed read (fragment) length and PSG $G = (V, E)$, if $\forall v \in V$, $rank(M^v) = outdegree(v), \forall \alpha$, then the model is identifiable.*

Proof. In the RNA-Seq model, each read is IID. Therefore, to show that the model is identifiable, we only need to show that if $P(r|\alpha) = P(r|\alpha'), \forall r$, then $\alpha = \alpha'$. Suppose we have a PSG that satisifies the conditions stated in the proposition. We prove by induction that $\alpha = \alpha'$ using a topological ordering of V. Suppose that for all vertices following v_k in the topological ordering, the weights of their outgoing edges are the same with α and α'. This is clearly the case for the last vertex in the ordering (the end vertex), which does not have any outgoing edges. Because the weights of all outgoing edges of vertices downstream of v_k are the same under α and α', the dominated-read matrices for v_k must also be identical. Given that $P(r|\alpha) = P(r|\alpha'), \forall r$, we must also have that $\mathbf{P}^{v_k}_{\alpha} = \mathbf{P}^{v_k}_{\alpha'} = \mathbf{P}^{v_k}$. Therefore,

$$M^{v_k}(D(\alpha)^{-1} f_\alpha(0,k)\boldsymbol{\alpha}_k) = M^{v_k}(D(\alpha')^{-1} f_{\alpha'}(0,k)\boldsymbol{\alpha'}_k) = \mathbf{P}^{v_k}$$

Since $rank(M^{v_k}) = outdegree(v)$, there must be a unique solution to the equation $M^{v_k}\mathbf{a} = \mathbf{P}^{v_k}$, and thus

$$D(\alpha)^{-1} f_\alpha(0,k)\boldsymbol{\alpha}_k = D(\alpha')^{-1} f_{\alpha'}(0,k)\boldsymbol{\alpha'}_k$$

Since the sums of the entries in $\boldsymbol{\alpha}_k$ and $\boldsymbol{\alpha'}_k$ are equal to one, we must have that $\boldsymbol{\alpha}_k = \boldsymbol{\alpha'}_k$. Therefore, the inductive hypothesis is true and we must have that $\alpha = \alpha'$. ☐

This proposition provides a general criterion for the identifiability of a PSG model. In the case of an unfactorized PSG, the only vertex with outdegree greater than one is the start vertex, and its outdegree is equal to the number of full-length isoforms. Thus this proposition reduces to the criterion described in (Lacroix ***et al.***, 2008; Hiller ***et al.***, 2009) in the case of an unfactorized PSG.

Unfortunately, it is difficult, for general non-unfactorized PSGs, to determine if $rank(M^v) = outdegree(v), \forall \alpha$. However, there are some specific cases for which it is easy to prove that this is the case. Proposition 1 in the main text provides one such useful case. We now provide the proof of this proposition.

Proof of Proposition 1 in the Main Text. We show that if $\forall (v, u) \in E$, there is a read that is uniquely derived from either (v, u), or $indegree(u) = 1$ and there is a read

uniquely derived from (u), then $rank(M^v) = outdegree(v), \forall v, \alpha$. Consider the reads dominated by vertex v_k with $outdegree(v_k) = n$. For each child, u_j of v_k, there must be a read that is uniquely derived from (v_k, u_j) or (u_j). The dominated-read vector for this read has a one in entry j and zeros for all other entries (irrespective of α). Therefore, M^{v_k} contains the $n \times n$ identity matrix as a submatrix and $rank(M^{v_k}) = n$. □

4 Computing MAP estimates of edge weights with EM

For simplicity of presentation, we again focus on the fixed-length single-end read model. MAP estimation requires computing

$$\arg\max_{\alpha} P(\alpha|r) = \arg\max_{\alpha} P(\alpha, r) \tag{1}$$

The marginal probability $P(\alpha, r)$ is

$$P(\alpha, r) = P(\alpha)P(r|\alpha) \tag{2}$$

$$= \left(C(\beta) \prod_{ij} \alpha_{ij}^{\beta_{ij}} \right) \left(D(\alpha)^{-N} \prod_{n=1}^{N} \sum_{(b,s)\in\pi(r)} \sum_{t:s\in t} w_\alpha(t) \right) \tag{3}$$

where

$$C(\beta) = \prod_i C_i(\beta)$$

$$C_i(\beta) = \frac{\Gamma(\sum_j (\beta_{ij} + 1))}{\prod_j \Gamma(\beta_{ij} + 1)}$$

is a constant with respect to α, so we will generally not be concerned with it.

Because the reads are the only observed random variables, $P(\alpha, r)$ involves a sum over all possible alignments for a read and all transcript paths that are compatible with those alignments. As $w_\alpha(t)$ is a function of α, this function is difficult to optimize directly. Therefore, we use the EM algorithm to perform this optimization, as is common for models with large numbers of latent variables. Unfortunately, since it is currently unknown whether Equation 2 is concave, we are only guaranteed to find a local maximum with EM.

The EM algorithm is concerned with the complete data joint probability, which is

$$P(r, z, \alpha) = C(\beta)D(\alpha)^{-N} \prod_{i,j} \alpha_{i,j}^{z_{ij}+\beta_{ij}} \tag{4}$$

where $Z_{ij} = \sum_n Z_{nij}$ and Z_{nij} is an indicator random variable that takes value 1 if edge (i, j) is part of the transcript, T_n, that generated read n.

4.1 E-step

In the E-step, we calculate $c_{ij} = E_{\alpha^{(t)}}[Z_{ij}]$. The expected value of Z_{nij} is computed as

$$E[Z_{nij}] = \frac{\sum_{(b,s)\in\pi(r_n)} g(s,i,j)}{\sum_{(b,s)\in\pi(r_n)} g(s)} \tag{5}$$

where

$$\begin{aligned} g(s) &= f(0,s_1)w(s) \\ g(s,i,j) &= \begin{cases} f(0,s_1)w(s) & (i,j)\in s \\ f(0,i)\alpha_{ij}f(j,s_1)w(s) & \text{if } \exists \text{ path from } v_j \text{ to } s_1 \\ f(0,s_1)w(s)f(s_{|s|},i)\alpha_{ij} & \text{if } \exists \text{ path from } s_{|s|} \text{ to } v_i \\ 0 & \text{otherwise} \end{cases} \end{aligned}$$

Note that Equation 5 takes into account multiple possible alignments of each read to the PSG. Because our methods currently perform inference on each PSG (gene) separately, a read that aligns to multiple genes (a "gene multiread") is included in the analysis for every gene to which it aligns.

For efficient computation, the $f(i,j)$ values and path weights $w(s)$ for each alignment may be precomputed at the beginning of the E-step. These two sets of values require $O(|V|^2)$ and $O(|A||E|)$ time to compute, respectively, where A is the set of alignments of all reads. In addition, the existence of paths between any two vertices can be precomputed in $O(|V|^2)$ time using dynamic programming before EM is run. With these precomputed values, the computation of all $E[Z_{nij}]$ values then requires $O(|A||E|)$ time. In total, each iteration of the E-step takes $O(|V|^2 + |A||E|)$ time.

4.1.1 Handling sequencing error and paired-end data

The E-step requires a few modifications for models of sequencing error and paired-end reads. Including sequencing error, Equation 5 becomes

$$E[Z_{nij}] = \frac{\sum_{(b,s)\in\pi(r_n)} g(s,i,j)P(r_n|s,b,q_n)}{\sum_{(b,s)\in\pi(r_n)} g(s)P(r_n|s,b,q_n)} \tag{6}$$

Thus, the quality of an alignment, represented by the probability $P(r_n|s,b,q_n)$, acts as a weight within the E-step calculation. Although the quality scores, q_n, are identical for each alignment, (b,s), of a read, r_n, the positions at which mismatches occur in the alignment may differ, resulting in different values for $P(r_n|s,b,q_n)$.

Additionally modeling paired-end data, Equation 5 becomes

$$E[Z_{nij}] = \frac{\sum_{(b,s,f)\in\pi(r_n)} g(s,i,j)P(r_n|s,b,q_n)P(f)}{\sum_{(b,s,f)\in\pi(r_n)} g(s)P(r_n|s,b,q_n)P(f)} \tag{7}$$

where the alignment of a read (pair) is represented as a triple (b,s,f), giving the start position of the upstream read, the subpath traversed by the fragment, and the fragment length implied by the alignment, respectively.

To obtain the possible alignments for a read pair, we first align each read separately and then compute all possible subpaths between an alignment of the first read and an alignment of the second read. In general, there may be an exponential number of possible subpaths between two read pair alignments. However, with typical RNA-Seq fragment lengths (~ 200 bases), the number of subpaths is usually small and not dependent on the total number of vertices in the graph.

One special case arises when one read of the pair aligns completely to the poly(A) tail, which is represented by the last vertex in a PSG. Because we don't know where in the tail the read aligns, there are many possible fragment lengths implied by this alignment. In these cases, the $P(f)$ term is replaced by $\int_{f_{min}}^{\infty} P(f)df$ in Equation 7, where f_{min} is the shortest possible fragment length, given the alignment of the non-poly(A) read.

4.2 M-step

The Q function for EM is

$$Q(\alpha|\alpha^{(t)}) = \log(C(\beta)) - N\log(D(\alpha)) + \sum_{i,j}(c_{ij} + \beta_{ij})\log\alpha_{ij} \tag{8}$$

and the Lagrangian for maximizing Q subject to the constraints that the edge parameters are probabilities is

$$\Lambda(\alpha,\lambda) = \log(C(\beta)) - N\log(D(\alpha)) + \sum_{i,j}(c_{ij}+\beta_{ij})\log\alpha_{ij} - \sum_i \lambda_i\left(\sum_j \alpha_{ij} - 1\right) \tag{9}$$

Taking derivatives with respect to α_{ij} and setting to zero, we have

$$0 = \frac{\partial\Lambda}{\partial\alpha_{ij}} = -\frac{N}{D(\alpha)}f(i)(d_f(i,\alpha) + d_b(j,\alpha)) + \frac{c_{ij}+\beta_{ij}}{\alpha_{ij}} - \lambda_i \tag{10}$$

Unfortunately, it is difficult to directly solve for the maximizing values of α_{ij} (note that $d_p(i)$ and $d_q(j)$ are also functions of α). Thus, to solve for α_{ij} we use fixed point iteration. In the special case where $\beta_{ij} = 0, \forall i, j$ (ML estimation), it can be shown that $\lambda_i = 0, \forall i$, and the fixed point iteration acts on the equation

$$\alpha_{ij} = \frac{\frac{c_{ij}}{(d_p(i)+d_q(j))}}{\sum_k \frac{c_{ik}}{(d_p(i)+d_q(k))}} \tag{11}$$

Thus, the ML estimate for α_{ij} is directly proportional to the number of times the edge is used, and inversely proportional to the average length of a transcript containing that edge. During each iteration, the $d_p(i)$ and $d_q(j)$ values can be computed once using their dynamic programming recurrences, and thus each iteration requires only $O(|V| + |E|)$ time.

5 Data sets and methods for differential processing experiments

5.1 DP tests on real data

We used three data sets for our differential processing experiments. The first set consisted of all five *Drosophila* samples analyzed by RNA-Seq in one of the modENCODE studies (Cherbas *et al.*, 2011). Two of these samples (SRA accessions SRS002587 and SRS002588) are biological replicates of cell line CME-W1-Cl.8+ and the other three (SRS002589, SRS002591, and SRS002594) are all from different cell lines. The RNA-Seq data for these samples were paired-end with a read length of 37. The second set of samples was obtained from a study of transcriptional differences between HapMap individuals (Montgomery *et al.*, 2010). We selected four RNA-Seq samples from this study, two technical replicates (ERR009098 and ERR009100) from a CEU individual, and two technical replicates (ERR009101 and ERR009112) from a Yoruban individual. The RNA-Seq data for these samples were paired-end with a read length of 36. A third set was created from data generated by the labs of Dr. Barbara Wold and Dr. Richard Myers as part of the ENCODE project (The ENCODE Project Consortium, 2011) on human cell lines K562 and HUVEC. This data set was downloaded from the UCSC ENCODE DCC (Rosenbloom *et al.*, 2010) and consisted of four paired-end (2x75) read sets representing two biological replicates for each of the two cell lines. The files downloaded were
wgEncodeCaltechRnaSeqK562R2x75Il200FastqRd1Rep1V2.fastq.tgz
wgEncodeCaltechRnaSeqK562R2x75Il200FastqRd1Rep2V2.fastq.tgz
wgEncodeCaltechRnaSeqK562R2x75Il200FastqRd2Rep1V2.fastq.tgz
wgEncodeCaltechRnaSeqK562R2x75Il200FastqRd2Rep2V2.fastq.tgz
wgEncodeCaltechRnaSeqHuvecR2x75Il200FastqRd1Rep1.fastq.tgz
wgEncodeCaltechRnaSeqHuvecR2x75Il200FastqRd1Rep2.fastq.tgz
wgEncodeCaltechRnaSeqHuvecR2x75Il200FastqRd2Rep1.fastq.tgz
wgEncodeCaltechRnaSeqHuvecR2x75Il200FastqRd2Rep2.fastq.tgz
from the URL:
http://hgdownload.cse.ucsc.edu/goldenPath/hg19/encodeDCC/wgEncodeCaltechRnaSeq/
To reduce analysis time, we selected the first 10 million pairs of reads from each of the ENCODE sets for our experiments.

For gene models, we used FlyBase annotations as described in the main text for the comparisons between EM and JR. For the human samples we used the RefSeq gene annotation (Pruitt *et al.*, 2009), also preprocessed by `cuffcompare`. Because of the long-range information provided by paired-end data, exon PSGs were used in this analysis instead of line PSGs.

For the PSG analyses, alignments were obtained by running Bowtie v0.12.7 (Langmead *et al.*, 2009) with parameters `--best --strata -l 25 -a -m 200` against reference sequences generated by the `psg_prepare_reference.py` script included in our software package. The `psg_infer_frequencies.py` script, which runs EM on all PSGs, was provided with average fragment length parameters (learned by running Cufflinks) for each set of samples. The `psg_infer_diff_processing.py`

script was then run to make DP calls. For DP analyses, we set the pseudocount parameter to zero (ML estimation). DP calls were made with the gene-level likelihood ratio test described in the main text.

For the FDM and Cufflinks analyses, we generated alignments by running TopHat v2.0.2 (Trapnell *et al.*, 2009) on each sample with options `--bowtie1 -r --mate-std-dev --bowtie-n --segment-length --no-novel-indels --no-novel-juncs -G -T` and given the appropriate parameter values for each sample. Using the TopHat alignments, Cuffdiff v2.0.0 was run to generate DP calls with options `-v --min-reps-for-js-test 1`.

The latest version of FDM (11-Aug-2011, as of the writing of this paper) was also used with the TopHat alignments to generate DP calls. FDM was run with configuration variables:

```
FDM_gene_file=all
fdm_inc_Novel=0
fdm_inc_start_end=0
fdm_partition=30
fdm_permutation=1000
fdm_pair_pvalue=0.05
filt_min_cov=0.01
filt_min_fdm=0.0
fdm2_permutation=1000
fdm2_pvalue=0.05
```

This is the default configuration for FDM, as determined from the example configuration files provided with its distribution, with the exception of the `fdm_inc_Novel=0` setting which instructs FDM to only consider known splice junctions. This modification to the default setting allowed us to compare the three DP methods using only annotated gene structures.

In order to run FDM without errors on all of our data sets, we had to fix one bug within its code. Specifically, on line 543 of of `fdm_all.py`, we had to change the statement

```
key_list = col_func(col_list,islandidxlist,islanddict)
```

to

```
key_list = set(col_func(col_list,islandidxlist,islanddict))
```

We communicated this bug and its fix to the author of the FDM software. In addition, for the fly data sets, we had to rename the *Drosophila* chromosomes to “chr1”, “chr2”, etc. in both the annotation files and the alignments to allow FDM to run without errors, as it appears to have been originally designed for mammalian genomes.

We ran the PSG, FDM and Cuffdiff DP tests on all pairs of samples within the two sets and computed the number of DP genes between them, using a false-discovery rate of 0.05. DP includes both differential splicing and differential transcription start site use, which are considered together by the PSG method but separately by Cuffdiff. Thus, we merged the two sets of Cuffdiff estimates into one set of DP calls.

5.2 DP tests on simulated data

We simulated four RNA-Seq data sets, two replicates from two biological conditions, using the `rsem-simulate-reads` simulator from the RSEM software package (Li and Dewey, 2011). The simulation parameters for each sample were learned using RSEM with standard options from one of the four human ENCODE samples analyzed in the previous section. Specifically, the simulation parameters for A Rep 1, A Rep 2, B Rep 1, and B Rep 2 were learned from HUVEC Rep 1, HUVEC Rep 2, K562 Rep 1, and K562 Rep 2, respectively. These parameters included those specifying gene abundances, sequencing error probabilities, and fragment length distributions.

Keeping the gene abundances the same as they were learned from each sample, we modified the simulation parameters to control the relative isoform frequencies of each gene and, correspondingly, the set of genes that were truly DP. To do this, we first constructed a single reference profile of relative isoform frequencies by picking the relative isoform frequencies for each gene from the learned parameters for one of the human ENCODE samples. Specifically, for each gene, we used the estimated relative isoform frequencies from the first sample that had non-zero expression of that gene, with a sample ordering of HUVEC Rep 1, HUVEC Rep 2, K562 Rep 1, and K562 Rep 2. The relative isoform frequencies for A Rep 1 and A Rep 2 were then set to those in this reference profile. Thus, no genes were truly DP between A Rep 1 and A Rep 2. We then constructed a modified profile for the B samples by randomly shuffling the relative isoform frequencies of 10% of the multi-isoform genes that had non-negligible expression (TPM $\geq$ 1) across all samples. We guaranteed that the randomly shuffled frequencies were different from those of the reference profile and therefore all of the selected genes were truly DP between condition A and B. The relative isoform frequencies for both B samples were set to those in the modified profile, and thus there were no truly DP genes between the two B replicates.

DP tests with PSG, FDM, and Cuffdiff were run on these simulated data with the same setting as for the real data. The DP predictions from each method with a target FDR of 0.05 were then compared to the true DP gene set to compute recall and precision. By varying the p-value threshold at which genes were called DP, we generated precision-recall curves (Figure S6). Since Cuffdiff provides several p-values for differential processing of each gene (one per isoform and one for differential promoter usage), we selected the minimum of these p-values as the DP score of each gene.

For multiple-replicate DP tests we provided all four simulated samples at the same time to both Cuffdiff and FDM, with A Rep 1 and A Rep 2 grouped as condition A, and B Rep 1 and B Rep 2 grouped as condition B. Accuracy measures were computed as for the tests without replication (Table S3). To produce comparable A vs. B accuracy measures for the PSG method, we took the mean of its accuracy measures over all pairs of non-replicate samples. Precision-recall curves were also generated (Figure S7), with the PSG curve generated by taking the mean of the precision-recall curves over all pairs of non-replicate samples. For the multiple replicate case, FDM does not provide p-values, so we instead varied a threshold for its reported "sig_difference" statistic to produce its precision-recall curve. This is equivalent to varying a p-value threshold because FDM internally computes a p-value using this statistic.

5.3 Testing the impact of the RNA-Seq read alignments on DP accuracy

To test if the different RNA-Seq read alignments used by the methods had an impact on our DP experiments, we devised an alternative to the TopHat alignments used for Cuffdiff and FDM that would be more comparable to the alignment used by the PSG method. The alternative alignment strategy was to align the RNA-Seq reads directly to the annotated transcript sequences (instead of to the genome) and then transform the resulting alignments to genomic coordinates so that they may be used by Cuffdiff and FDM. To implement this strategy, we first used RSEM's `rsem-prepare-reference` script with its `--no-polyA` option to construct transcript sequences from the given gene annotation and reference genome. Paired-end read data were then aligned to these sequences using Bowtie with options `--best`, `--strata`, `-l 25`, `-a`, `-m 200`, and `-X 1000` to best match the alignments used by the PSG method. These transcript-based alignments were then transformed to genomic coordinates using RSEM's `rsem-tbam2gbam` program. The results of using these alternative alignments with Cuffdiff and FDM are shown in Table S2 and Figure S6.

6 Supplementary tables

Sample 1	Sample 2	PSG	FDM	Cuffdiff	PSG ∩ FDM	PSG ∩ Cuffdiff	FDM ∩ Cuffdiff	All
**HUVEC Rep 1**	**HUVEC Rep 2**	**73**	**32**	**474**	**6**	**9**	**2**	**1**
HUVEC Rep 1	K562 Rep 1	448	201	7	71	4	0	0
HUVEC Rep 1	K562 Rep 2	384	137	6	36	4	0	0
HUVEC Rep 2	K562 Rep 1	492	228	8	81	2	0	0
HUVEC Rep 2	K562 Rep 2	414	156	10	48	2	0	0
**K562 Rep 1**	**K562 Rep 2**	**260**	**143**	**175**	**26**	**16**	**0**	**0**

Table S1: The number of DP genes called by the PSG test, FDM, Cuffdiff, and combinations of the methods on pairs of samples from the trimmed ENCODE set, in which the reads of each of the samples were trimmed to a length of 36 bp before analysis by all of the methods. Pairs of samples that are technical or biological replicates are indicated in bold.

Method	Sample 1	Sample 2	Predicted DP	Recall	Precision
PSG	**A Rep 1**	**A Rep 2**	**4**		
	A Rep 1	B Rep 1	257	0.60	0.95
	A Rep 1	B Rep 2	230	0.54	0.95
	A Rep 2	B Rep 1	251	0.59	0.94
	A Rep 2	B Rep 2	235	0.54	0.93
	**B Rep 1**	**B Rep 2**	**0**		
Cuffdiff	**A Rep 1**	**A Rep 2**	**379**		
	A Rep 1	B Rep 1	49	0.11	0.92
	A Rep 1	B Rep 2	58	0.13	0.88
	A Rep 2	B Rep 1	48	0.12	0.98
	A Rep 2	B Rep 2	51	0.11	0.88
	**B Rep 1**	**B Rep 2**	**148**		
FDM	**A Rep 1**	**A Rep 2**	**11**		
	A Rep 1	B Rep 1	311	0.39	0.51
	A Rep 1	B Rep 2	255	0.28	0.44
	A Rep 2	B Rep 1	320	0.37	0.47
	A Rep 2	B Rep 2	242	0.24	0.40
	**B Rep 1**	**B Rep 2**	**148**		
Cuffdiff (Bowtie)	**A Rep 1**	**A Rep 2**	**263**		
	A Rep 1	B Rep 1	39	0.08	0.85
	A Rep 1	B Rep 2	38	0.08	0.89
	A Rep 2	B Rep 1	31	0.07	0.90
	A Rep 2	B Rep 2	37	0.08	0.92
	**B Rep 1**	**B Rep 2**	**49**		
FDM (Bowtie)	**A Rep 1**	**A Rep 2**	**9**		
	A Rep 1	B Rep 1	317	0.35	0.45
	A Rep 1	B Rep 2	234	0.30	0.51
	A Rep 2	B Rep 1	320	0.36	0.45
	A Rep 2	B Rep 2	223	0.30	0.54
	**B Rep 1**	**B Rep 2**	**58**		

Table S2: The accuracy of the DP-calling methods on the simulated RNA-Seq data sets with a target FDR of 0.05. Pairs of replicates from same simulated biological condition are in bold and the genes predicted to be DP for these pairs are all considered to be false positives. FDM (Bowtie) and Cuffdiff (Bowtie) refer to the running of these methods with alignments using Bowtie directly to transcript sequences, rather than with alignments to the genome using TopHat.

Method	Predicted DP	Recall	Precision
PSG	243	0.57	0.94
Cuffdiff	232	0.49	0.86
FDM	0	0.00	1.00
Cuffdiff (Bowtie)	264	0.50	0.76
FDM (Bowtie)	0	0.00	1.00

Table S3: The accuracy of the DP-calling methods for a condition A vs. condition B test with the simulated RNA-Seq data. For this test, all replicates were provided to Cuffdiff and FDM at the same time. The accuracy measures reported for the PSG method are the means over all non-replicate pair tests (see Table S2). FDM (Bowtie) and Cuffdiff (Bowtie) refer to the running of these methods with alignments using Bowtie directly to transcript sequences, rather than with alignments to the genome using TopHat.

7 Supplementary figures

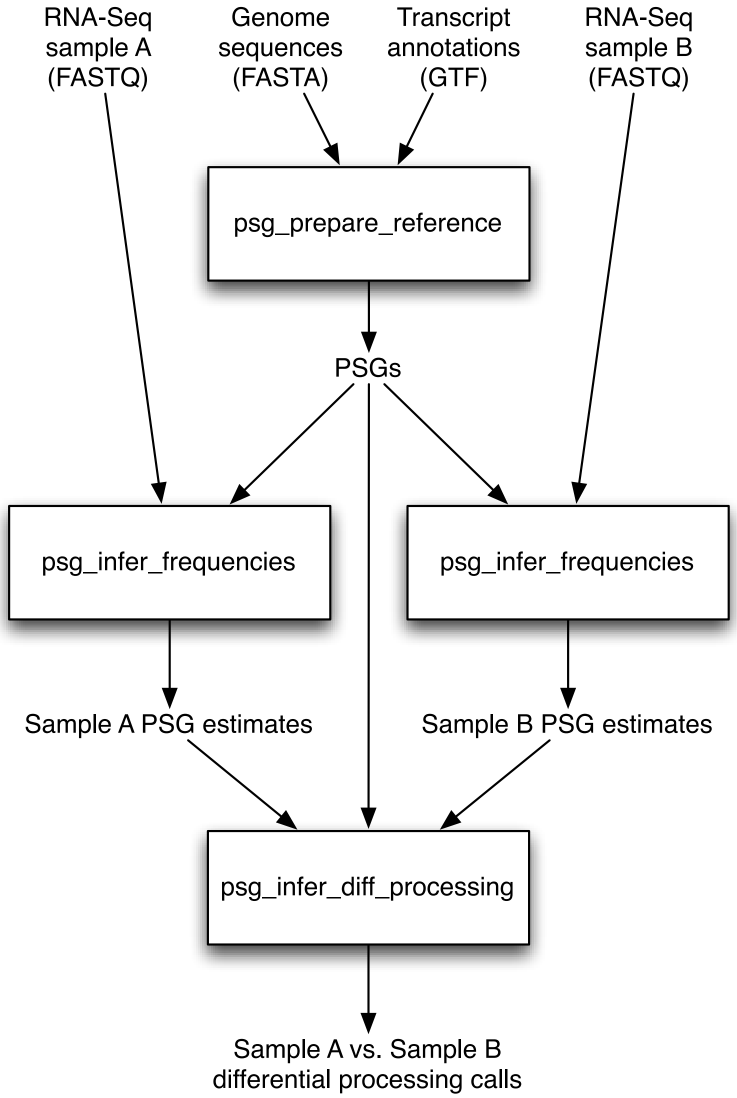

Figure S1: A typical workflow for running the PSGInfer scripts to detect differential processing between a pair of RNA-Seq samples. PSGs may also be constructed independently of a genome sequence and annotation (e.g., from a *de novo* transcriptome assembly).

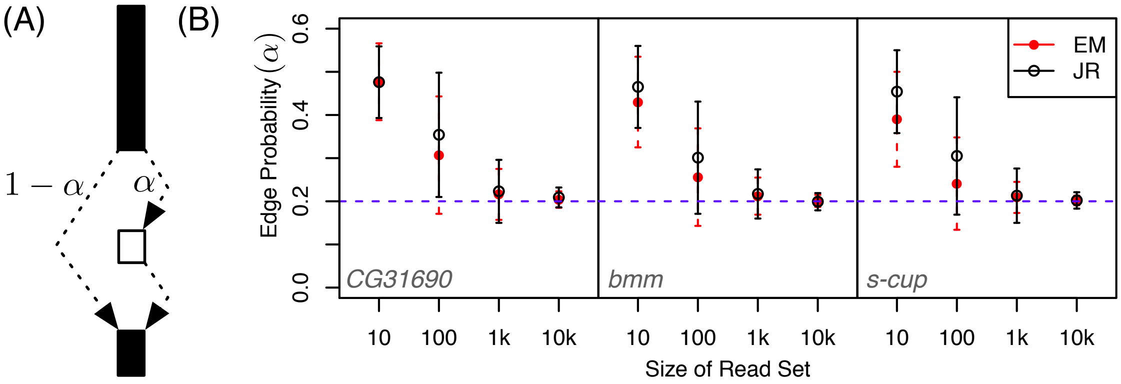

Figure S2: Parameter estimates for the EM approach converge more quickly and are less variable than those of JR. Error-free RNA-Seq read sets were simulated from three fly genes (*CG31690*, *bmm*, and *s-cup*), each of which contain a single cassette exon as depicted by the gene model in (A). The lengths of the cassette exons for the genes are 76, 136, and 253, respectively. The gene model has one parameter, α, which is the probability of inclusion of the cassette exon. The mean (with s.d. bars) of the estimates for the simulations of a given read set size are shown in (B). For all simulations, $\alpha = 0.20$ (dotted blue line).

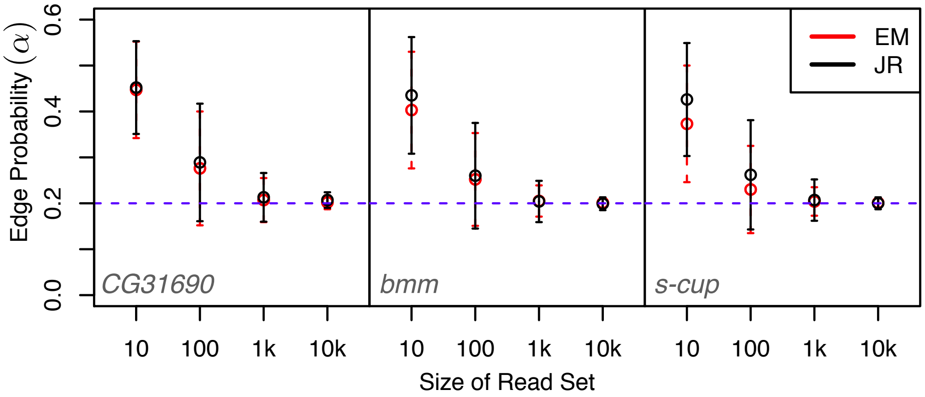

Figure S3: Convergence and variation of parameter estimates for EM and JR on simulated paired-end read data, as described in Section 3.1.1 of the main text.

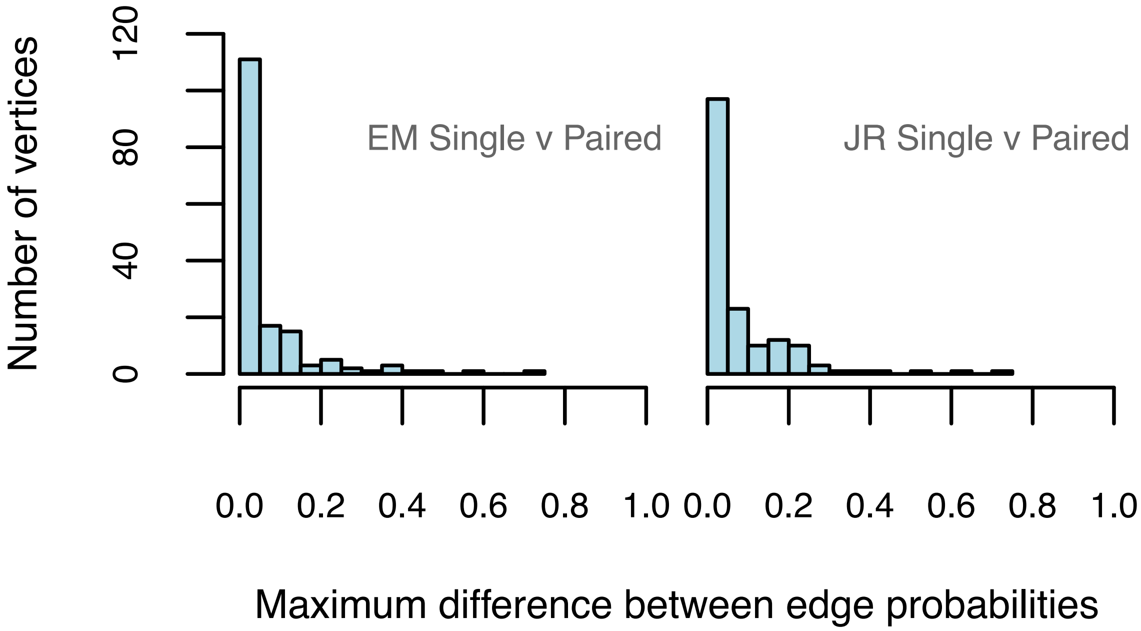

Figure S4: Distributions of the differences between the parameter estimates from the same method (either EM or JR) on single and paired-end data, as described in Section 3.1.2 of the main text.

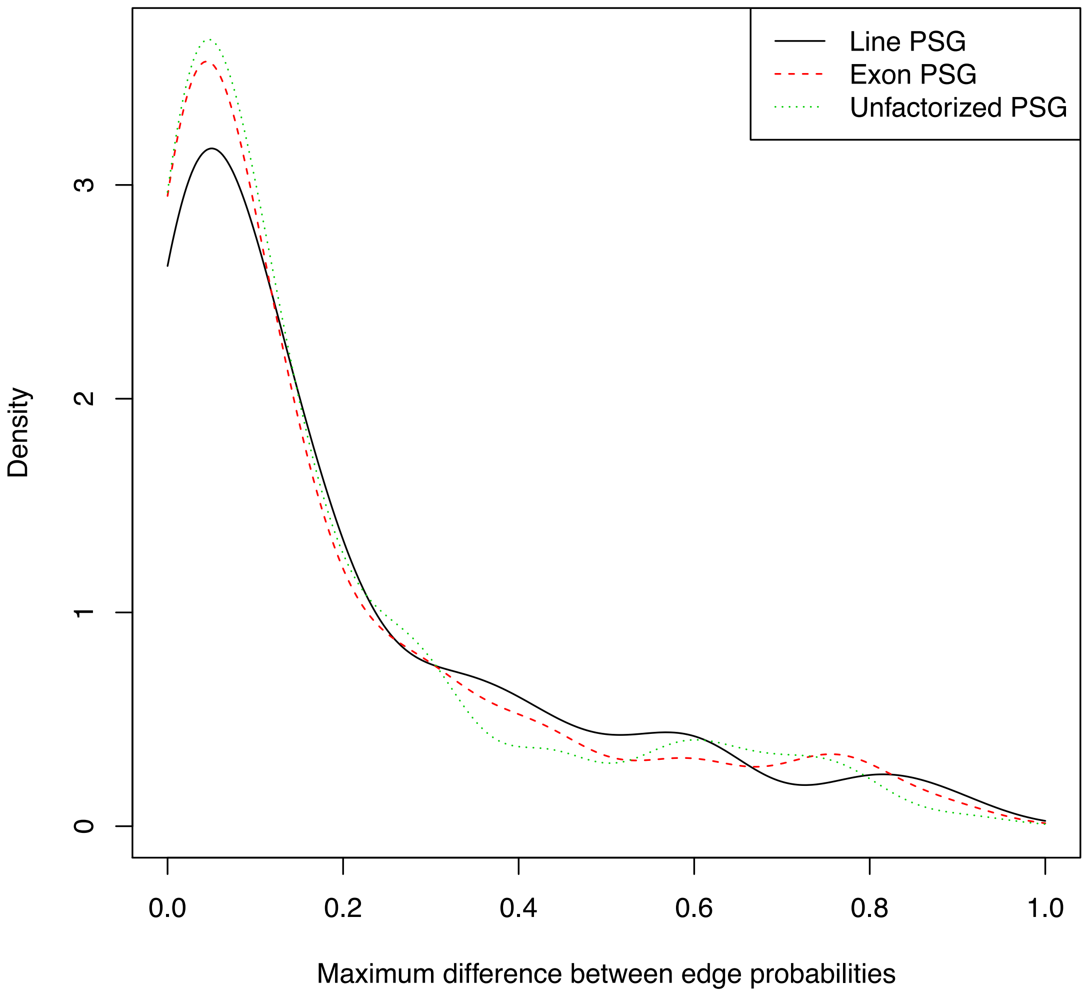

Figure S5: Distributions of the differences between the parameter estimates of EM to those of JR from paired-end data, for three different PSG models: line, first-order exon, and unfactorized, as described in Section 3.1.2 of the main text. None of the difference distributions are found to be significantly different from each other according to the Wilcoxon signed rank test (p-values of 0.15, 0.20, and 0.69, for line vs. first-order exon, line vs. unfactorized, and first-order exon vs. unfactorized, respectively).

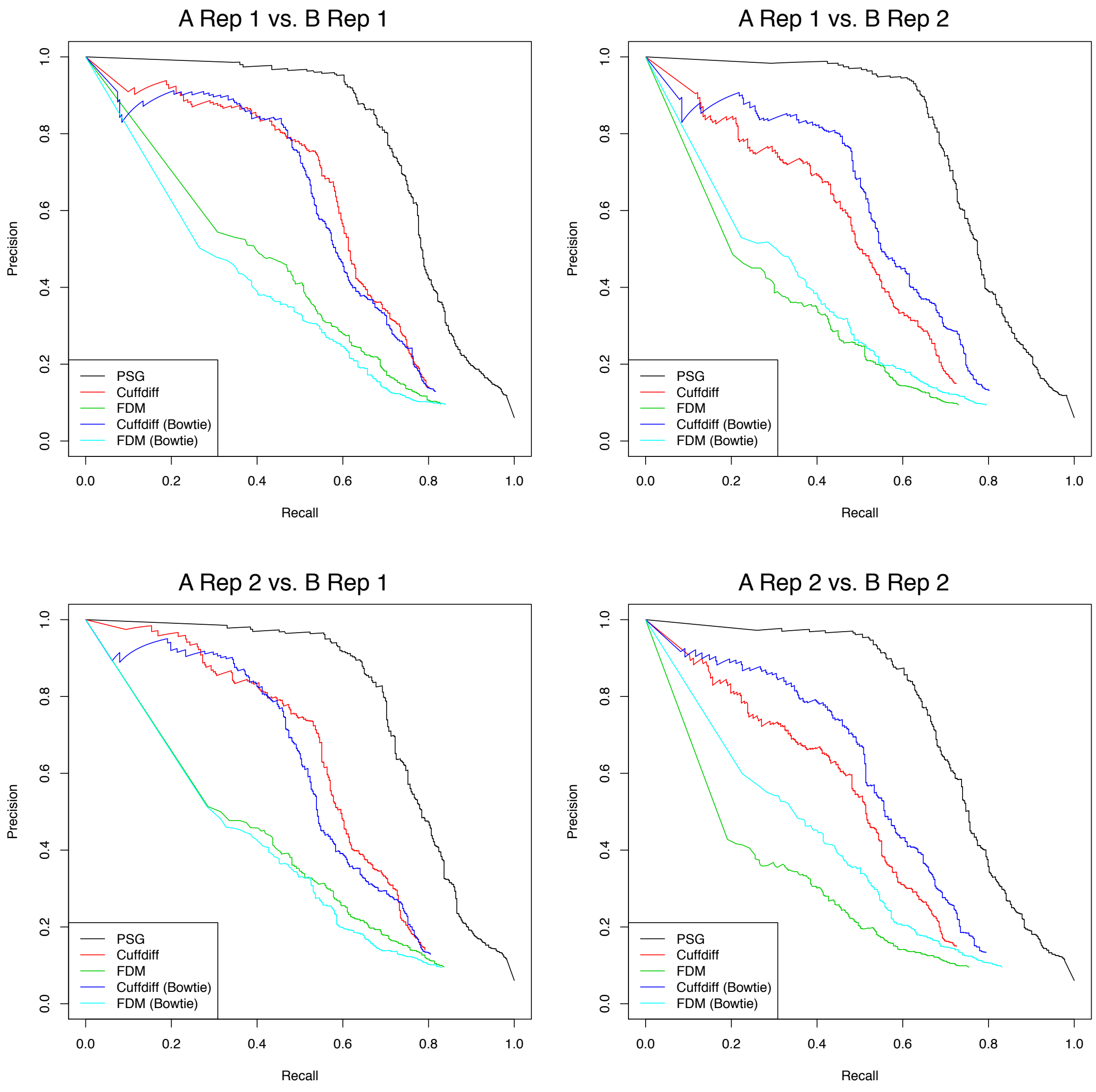

Figure S6: Precision-Recall curves for the DP-calling methods on the simulated RNA-Seq data sets. FDM (Bowtie) and Cuffdiff (Bowtie) refer to the running of these methods with alignments using Bowtie directly to transcript sequences, rather than with alignments to the genome using TopHat.

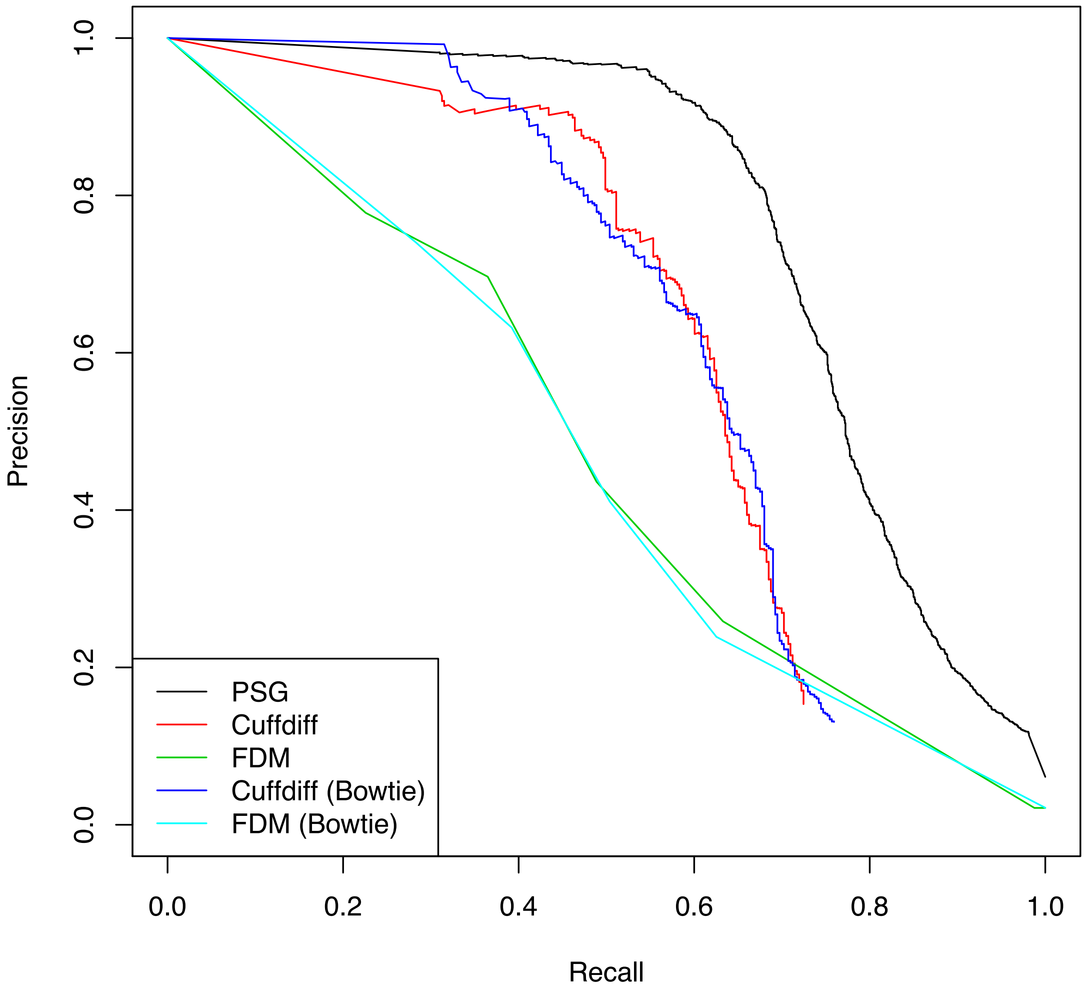

Figure S7: Precision-Recall curves for the DP-calling methods for a condition A vs. condition B test with the simulated RNA-Seq data. For this test, all replicates were provided to Cuffdiff and FDM at the same time. The precision-recall curve for the PSG method is the mean precision-recall curve over all non-replicate pair tests (see Figure S6). FDM (Bowtie) and Cuffdiff (Bowtie) refer to the running of these methods with alignments using Bowtie directly to transcript sequences, rather than with alignments to the genome using TopHat.

References

Cherbas, L., Willingham, A., Zhang, D., Yang, L., Zou, Y., Eads, B. D., Carlson, J. W., Landolin, J. M., Kapranov, P., Dumais, J., Samsonova, A., Choi, J.-H., Roberts, J., Davis, C. a., Tang, H., van Baren, M. J., Ghosh, S., Dobin, A., Bell, K., Lin, W., Langton, L., Duff, M. O., Tenney, A. E., Zaleski, C., Brent, M. R., Hoskins, R. a., Kaufman, T. C., Andrews, J., Graveley, B. R., Perrimon, N., Celniker, S. E., Gingeras, T. R., and Cherbas, P. (2011). The transcriptional diversity of 25 Drosophila cell lines. *Genome Research*, **21**(2), 301–14.

Hiller, D., Jiang, H., Xu, W., and Wong, W. H. (2009). Identifiability of isoform deconvolution from junction arrays and RNA-Seq. *Bioinformatics*, **25**(23), 3056–9.

Lacroix, V., Sammeth, M., Guigo, R., and Bergeron, A. (2008). Exact transcriptome reconstruction from short sequence reads. *Algorithms in Bioinformatics*, **5251**, 50–63.

Langmead, B., Trapnell, C., Pop, M., and Salzberg, S. L. (2009). Ultrafast and memory-efficient alignment of short DNA sequences to the human genome. *Genome Biology*, **10**(3), R25.

Li, B. and Dewey, C. N. (2011). RSEM: accurate transcript quantification from RNA-Seq data with or without a reference genome. *BMC Bioinformatics*, **12**(1), 323.

Montgomery, S. B., Sammeth, M., Gutierrez-Arcelus, M., Lach, R. P., Ingle, C., Nisbett, J., Guigo, R., and Dermitzakis, E. T. (2010). Transcriptome genetics using second generation sequencing in a Caucasian population. *Nature*, **464**(7289), 773–7.

Pruitt, K. D., Tatusova, T., Klimke, W., and Maglott, D. R. (2009). NCBI Reference Sequences: current status, policy and new initiatives. *Nucleic Acids Research*, **37**(Database issue), D32–6.

Rosenbloom, K. R., Dreszer, T. R., Pheasant, M., Barber, G. P., Meyer, L. R., Pohl, A., Raney, B. J., Wang, T., Hinrichs, A. S., Zweig, A. S., Fujita, P. A., Learned, K., Rhead, B., Smith, K. E., Kuhn, R. M., Karolchik, D., Haussler, D., and Kent, W. J. (2010). ENCODE whole-genome data in the UCSC Genome Browser. *Nucleic Acids Research*, **38**(Database issue), D620–5.

The ENCODE Project Consortium (2011). A User's Guide to the Encyclopedia of DNA Elements (ENCODE). *PLoS Biology*, **9**(4), e1001046.

Trapnell, C., Pachter, L., and Salzberg, S. L. (2009). TopHat: discovering splice junctions with RNA-Seq. *Bioinformatics*, **25**(9), 1105–11.
